# The Golgi stacking protein GRASP55 is targeted by the natural compound prodigiosin

**DOI:** 10.1186/s12964-023-01275-1

**Published:** 2023-10-05

**Authors:** Lena Berning, Thomas Lenz, Ann Kathrin Bergmann, Gereon Poschmann, Hannah U. C. Brass, David Schlütermann, Annabelle Friedrich, María José Mendiburo, Céline David, Seda Akgün, Jörg Pietruszka, Kai Stühler, Björn Stork

**Affiliations:** 1 Institute of Molecular Medicine I, Medical Faculty and University Hospital Düsseldorf, Heinrich Heine University, Düsseldorf, 40225 Germany; 2 Molecular Proteomics Laboratory, Biological Medical Research Centre, Heinrich Heine University, 40225 Düsseldorf, Germany; 3 Core Facility for Electron Microscopy, Medical Faculty and University Hospital Düsseldorf, Heinrich Heine University, Düsseldorf, 40225 Germany; 4 Institute of Molecular Medicine I, Proteome Research, Medical Faculty and University Hospital Düsseldorf, Heinrich Heine University, Düsseldorf, 40225 Germany; 5 Institute of Bioorganic Chemistry, Heinrich Heine University Düsseldorf at Forschungszentrum Jülich and Bioeconomy Science Center (BioSC), 52426 Jülich, Germany; 6 Institute of Bio- and Geosciences: Biotechnology (IBG-1), Forschungszentrum Jülich, 52428 Jülich, Germany

**Keywords:** Prodigiosin, Golgi apparatus, Natural compound, Autophagy, Target identification

## Abstract

**Background:**

The bacterial secondary metabolite prodigiosin has been shown to exert anticancer, antimalarial, antibacterial and immunomodulatory properties. With regard to cancer, it has been reported to affect cancer cells but not non-malignant cells, rendering prodigiosin a promising lead compound for anticancer drug discovery. However, a direct protein target has not yet been experimentally identified.

**Methods:**

We used mass spectrometry-based thermal proteome profiling in order to identify target proteins of prodigiosin. For target validation, we employed a genetic knockout approach and electron microscopy.

**Results:**

We identified the Golgi stacking protein GRASP55 as target protein of prodigiosin. We show that prodigiosin treatment severely affects Golgi morphology and functionality, and that prodigiosin-dependent cytotoxicity is partially reduced in GRASP55 knockout cells. We also found that prodigiosin treatment results in decreased cathepsin activity and overall blocks autophagic flux, whereas co-localization of the autophagosomal marker LC3 and the lysosomal marker LAMP1 is clearly promoted. Finally, we observed that autophagosomes accumulate at GRASP55-positive structures, pointing towards an involvement of an altered Golgi function in the autophagy-inhibitory effect of this natural compound.

**Conclusion:**

Taken together, we propose that prodigiosin affects autophagy and Golgi apparatus integrity in an interlinked mode of action involving the regulation of organelle alkalization and the Golgi stacking protein GRASP55.

Video Abstract

**Supplementary Information:**

The online version contains supplementary material available at 10.1186/s12964-023-01275-1.

## Background

Historically, natural products have played a key role in drug discovery. Especially for cancer and infectious diseases, nature-derived compounds make up a considerable proportion of medication [52]. Stimulated by evolutionary pressure, plants, fungi and microorganisms can produce an almost inexhaustible diversity of bioactive compounds. These compounds often display a complex structure and stereochemistry, which—in some cases—can hardly be mimicked by synthetic approaches [12]. However, natural compounds have their pitfalls when it comes to disposability, purity, and bioavailability. To overcome these drawbacks, semi-synthetic approaches allowed by advances in genomics, bioinformatics and replicating synthesis have been deployed. Natural products can target miscellaneous molecular pathways in eukaryotic cells and though various methods for target identification have been developed in the previous decades, it remains challenging to identify the molecular targets of these often highly bioactive metabolites due to widely varied mechanisms of action and diverse and often multiple targets [9]. Knowing the mechanisms of action of natural products can pave the way to the discovery of new targets and cellular pathways with high specificity towards cancerous cells in order to fill the enormous need for new therapeutic options caused by therapeutic failure as a result of drug resistance or relapse.

In recent years, the natural compound prodigiosin has been shown to exert promising biomedical activities. It is a deeply red secondary metabolite with a tripyrrole structure (reviewed in [22, 27]). Although 'optically' known for centuries, prodigiosin was first extracted from *Serratia marcescens* by Wrede and Hettche in 1929 [75], followed by partial and total syntheses in the 1960s [60, 73]. In addition to the extraction from various bacterial strains, prodigiosin and its analogues can be produced via semi-synthetic und synthetic approaches [20, 35, 36, 45, 79]. Prodigiosin has been shown to possess various beneficial effects like anticancer [74], antimalarial [8] and antimicrobial [13] properties. The anticancer properties of this natural compound have so far been linked to the modulation of autophagy [10, 35, 36], lysosomal activity [82] or apoptosis [30]. Mechanistically, prodigiosin has been described as an H^+^/Cl^−^ symporter, which can lead to an alkalization of acidic organelles such as endosomes, lysosomes or the Golgi apparatus by uncoupling the vacuolar-type H^+^-ATPase (V-ATPase) [61, 63].

The Golgi apparatus is an essential organelle located in the perinuclear region of mammalian cells [39]. As a receiver of the majority of the endoplasmic reticulum (ER) output, the Golgi apparatus acts as the central hub for post-translational modifications and sorting of proteins and lipids for the secretory pathway [5]. For its proper functioning, the Golgi is organized in stacks of flattened cisternae that are often laterally linked into a ribbon-like structure. The only proteins that have been shown to be responsible for establishing the stacked structure of the Golgi so far are the Golgi reassembly stacking proteins of 55 kDa (GRASP55, primary gene name: GORASP2) and of 65 kDa (GRASP65, primary gene name: GORASP1), which are localized to the *trans* and *cis* cisternae, respectively [4, 64]. These GRASP proteins are peripheral membrane proteins which form trans-oligomers from adjacent cisternae to link the Golgi stacks into a ribbon [58, 71, 76]. In addition to its role in Golgi stacking, GRASP55 has been previously described to be involved in unconventional secretion [1, 53] and autophagy [43, 80, 81].

Autophagy is an intracellular catabolic process in which misfolded, damaged or aggregated proteins as well as whole cell organelles can be degraded and recycled (reviewed in [18, 78]). Late stage autophagy is dependent on autophagosome-lysosome fusion and the hydrolase activity of lysosomal enzymes for the breakdown of the autophagic cargo. The above described alkalization of lysosomes and the resulting inhibition of pH-dependent lysosomal hydrolases likely represent one mechanism of prodigiosin-mediated autophagy inhibition. In addition to the alkalization of lysosomes, prodigiosin has previously been described to block the fusion of autophagosome and lysosome [82]. The potent autophagy-inhibitory property of prodigiosin has also been reported in a previous work from our groups, in which we observed that prodigiosin re-sensitized cisplatin-resistant cells to apoptotic cell death [6]. Furthermore, prodigiosin derivatives have been identified that displayed more potent autophagy inhibitory activity than the parent compound or the synthetic derivative obatoclax [35, 36]. However, no molecular target of prodigiosin has been identified so far.

A commonly used technique for target engagement is the cellular thermal shift assay (CETSA) which is based on the principle of thermal stabilization of an intracellular protein when it is bound to a small molecule [29, 46]. The temperature at which denaturation and irreversible precipitation of a protein occurs within thermally treated cells can be shifted by complexation with a ligand and the non-denatured, soluble protein fraction can then be investigated by immunoblotting. A huge advantage of this method is the possibility to assess drug-protein interactions under physiological conditions in living cells without labelling or immobilization of the compound or the protein of interest. To analyze several thousands of proteins in an unbiased approach, CETSA can be combined with multiplexed quantitative MS analysis. This approach for identifying novel protein targets of small molecules in living cells has been termed thermal proteome profiling (TPP) [21, 62]. Recently, the TPP technique has been extended so that, in addition to protein thermal stability alternation, differences in protein abundance can also be detected in the same experiment, termed ratio-based thermal shift assay analysis (RTSA) [33].

In this study, we used the TPP/RTSA approach to identify protein targets and other affected proteins of the natural compound prodigiosin. We found that prodigiosin thermally stabilizes the Golgi protein GRASP55, affects lysosomal proteins as well as proteins involved in autophagy, and that prodigiosin treatment severely alters the structure of the Golgi apparatus while the knockout of GRASP55 partly reverses prodigiosin cytotoxicity. We also observed that autophagosomes accumulate at the Golgi apparatus while overall autophagic flux is inhibited in cells treated with prodigiosin. Thus, we propose GRASP55 as a target protein of prodigiosin.

## Results

### Prodigiosin exhibits cytotoxic properties in HeLa cells and alters Golgi apparatus morphology

**Fig. 1:**
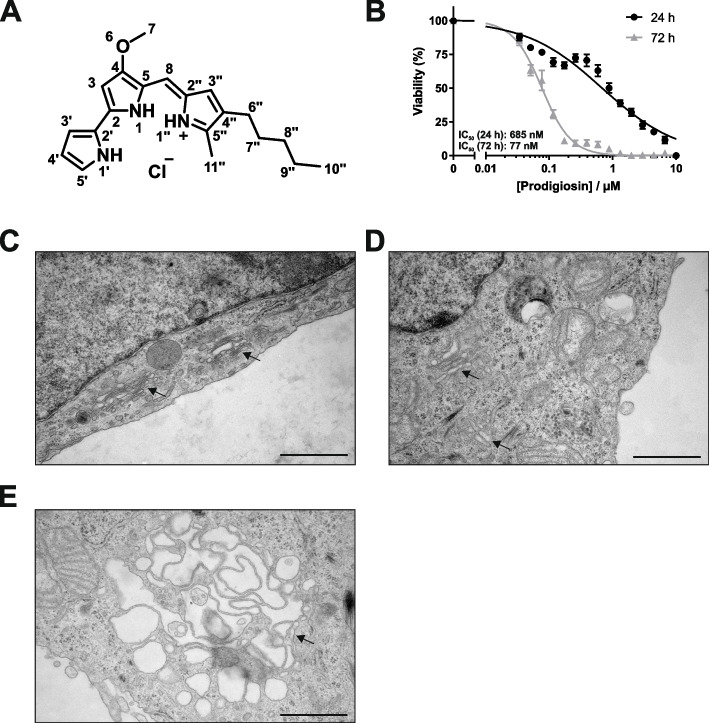
Prodigiosin exhibits cytotoxic properties in HeLa wt cells and alters Golgi apparatus morphology. **A** Chemical structure of prodigiosin. **B** HeLa wt cells were treated with different concentrations of prodigiosin for 24 h or 72 h. After treatment, cell viability was measured using a thiazolyl blue (MTT) assay. Results are shown as the mean ± SEM of three independent experiments performed in triplicates for each treatment. HeLa wt cells were treated with **C** DMSO, **D** 10 nM or **E** 100 nM prodigiosin for 24 h and effects on cell morphology were investigated by transmission electron microscopy. Arrows indicate changes in Golgi apparatus morphology after prodigiosin treatment. Representative electron micrographs are shown. Scale bar: 1 µm

Prodigiosin (Fig. 1A) has been shown to exert cytotoxic effects in various cancer cell lines [6, 30, 42]. In HeLa cells, prodigiosin was confirmed to be highly cytotoxic with IC_50_ values in the nanomolar range both after 24 h and 72 h (Fig. 1B). Aiming to identify the molecular mechanism of prodigiosin cytotoxicity, we examined if we could observe structural changes in cell morphology after prodigiosin treatment through transmission electron microscopy (TEM). We chose 10 and 100 nM prodigiosin and a treatment duration of 24 h, since cells remained mostly viable under these conditions. While we observed distinct Golgi stacks with multiple long and thin cisternae in vehicle (DMSO) treated cells (Fig. 1C), cisternae number and length decreased and cisternae were more voluminous upon treatment with 10 nM prodigiosin (Fig. 1D). After treatment with 100 nM prodigiosin, we observed a distinct swelling of the Golgi apparatus (Fig. 1E).

**Fig. 2:**
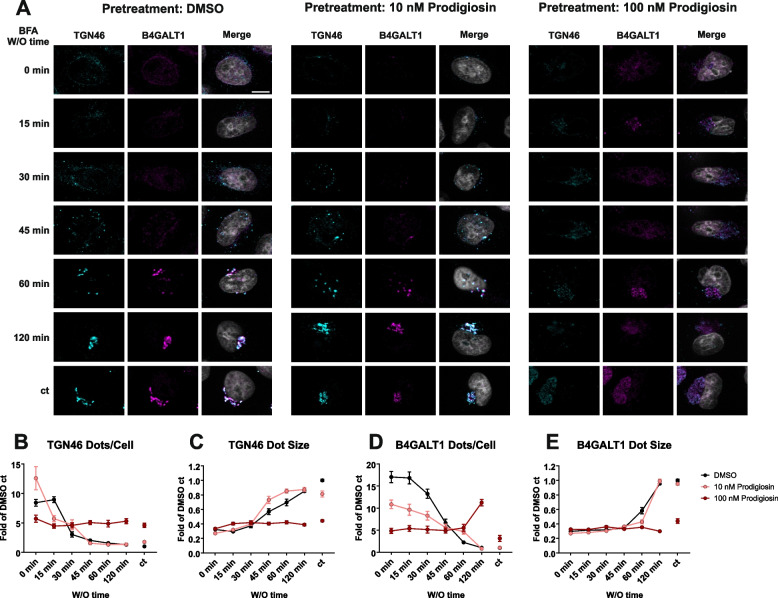
Pre-treatment with prodigiosin impairs Golgi apparatus reassembly after BFA treatment. HeLa wt cells were seeded on cover slips. On the next day, cells were treated with DMSO, 10 nM prodigiosin or 100 nM prodigiosin for 24 h. Cells were washed once and treated with 5 μg/mL brefeldin A (BFA) or DMSO (ct) for 2 h. BFA was washed out with DPBS 4 times and cells were incubated in fresh growth medium to wash out (W/O) BFA for 0/15/30/45/60/120 min. After treatment, cover slips were prepared for microscopy. **A** Representative sections are depicted. Scale bar: 10 µm. **B-E** The relative number per cell and mean area of TGN46 and B4GALT1 positive structures of 15 representative images from three biological replicates for each treatment were quantified using ImageJ 1.53c

To further examine potential prodigiosin effects on Golgi apparatus structure and function, we utilized the brefeldin A (BFA) washout assay. The fungal metabolite BFA inhibits ER to Golgi transport and causes Golgi disassembly and a reversible redistribution of Golgi cisternae into the ER [34]. The effects of BFA treatment can be reversed by removing the drug and allowing the Golgi apparatus to reassemble its structure. For the BFA washout experiment, HeLa cells were pre-treated with DMSO as a control or with 10 or 100 nM prodigiosin. Then, Golgi apparatus structure was disassembled by 2 h treatment with BFA. To monitor Golgi reassembly during BFA washout, we immunostained for the Golgi membrane protein *trans*-Golgi network glycoprotein 46 (TGN46) and the Golgi enzyme β-1,4-galactosyltransferase 1 (B4GALT1) (Fig. 2A) and quantified average size and number of stained structures (Fig. 2B-E). After incubation with BFA, normal Golgi structures (as determined by TGN46 or B4GALT1 staining) completely disappeared, and small structures with weak fluorescence signal were observed homogenously distributed over the cell. In cells pre-treated with DMSO or 10 nM prodigiosin, TGN46 structures started to reform 30 min after onset of Golgi regeneration. During ongoing BFA washout, less (Fig. 2B) but bigger (Fig. 2C) TGN46-positive structures rebuilt under these two conditions, representing the reassembly of the Golgi apparatus. After 120 min washout, TGN46 dot size, number and localization were similar to cells that had not been treated with BFA (ct, Fig. 2B and C). In contrast, no changes in dot size or number for TGN46 were observed during BFA washout in cells pre-treated with 100 nM prodigiosin (Fig. 2B and C). Directly after Golgi disassembly, cells pre-treated with 100 nM prodigiosin had less dots than cells pre-treated with DMSO, and the overall dot number remained constant over the 120 min period. Furthermore, no reassembly of larger structures was observed in cells pre-treated with 100 nM prodigiosin even after 120 min washout time, suggesting a severely disturbed Golgi reassembly. Similarly, in cells that were not exposed to BFA the typical dense perinuclear staining was not found, but instead a diffuse and more dispersed staining in the perinuclear region. Essentially, similar observations were made for the second marker protein, B4GALT1 (Fig. 2D and E): a decrease of dot number and an increase in dot size over time for cells pre-treated with DMSO or 10 nM prodigiosin indicating a fully reformed Golgi complex, but a rather constant number and size of dots in cells pre-treated with 100 nM prodigiosin (please note that the dot number even increased after 120 min of BFA washout). Taken together, we observed clear effects on Golgi structure and reassembly upon prodigiosin treatment.

### Thermal proteome profiling identifies GRASP55 as a target protein of prodigiosin

Taking these immense effects on the Golgi apparatus into account, we next aimed to identify the molecular target of prodigiosin to elucidate the mechanism of action of this potent natural compound. We chose the TPP approach [62] due to its ability for target protein identification and engagement in an unperturbed live cell system using non-derivatized prodigiosin (Fig. S1). TPP is based on the principle of thermal stabilization (or less frequently destabilization) of a protein when bound to a small molecule (or other ligands), resulting in altered protein melting characteristics, and exploits the differential protein denaturation and irreversible precipitation in the bound and unbound state upon thermal treatment. In complement to identify prodigiosin-induced thermal stability alternation of proteins, as is done with the classical TPP setup, we chose an experimental setup similar to that described [33], which allowed us to detect prodigiosin-affected differential protein abundance (e.g., by differential protein expression, degradation or secretion) as well.

**Fig. 3:**
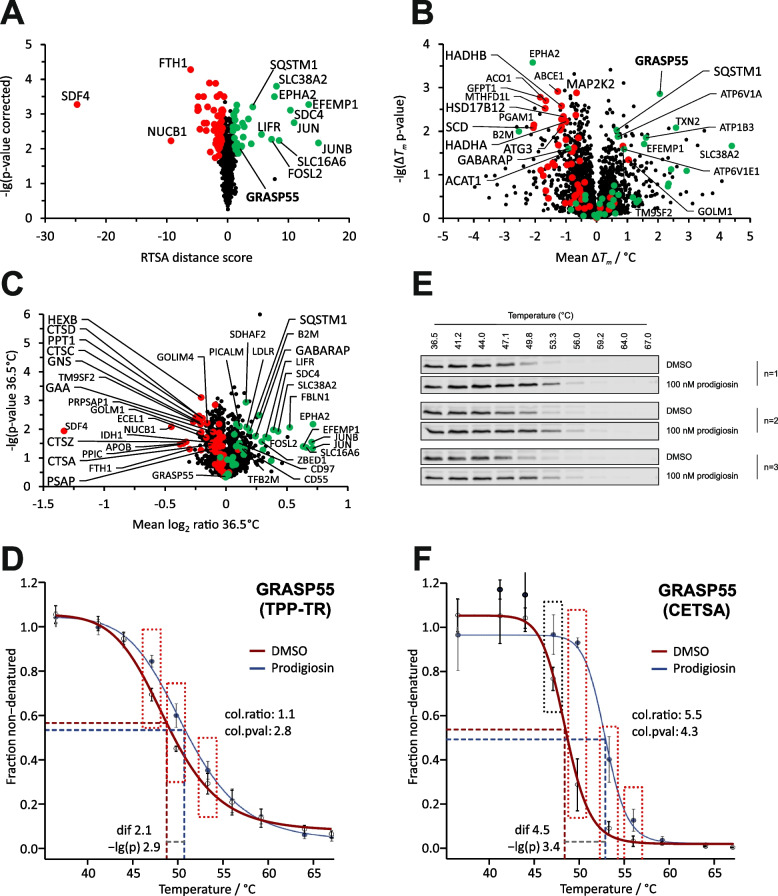
Thermal proteome profiling (TPP) for the identification of prodigiosin targets and prodigiosin-affected proteins: GRASP55 is thermally stabilized by treatment with prodigiosin. **A** RTSA analysis plot of the statistical significance vs. the extent of the effect of prodigiosin on protein intensity collated over the different temperatures. Differential protein intensities may result from prodigiosin-mediated thermal protein stability alternation and/or a change in protein abundance (caused by, e.g., differential protein expression, degradation or secretion), where these two effects cannot be distinguished in the current plot. Significant proteins given by the RTSA software are colored in green or red for positive or negative RTSA distance score, respectively. **B** Volcano-like plot of the statistical significance vs. the extent of prodigiosin-mediated thermal protein stabilization (mean Δ*T*_*m*_ > 0) or destabilization (mean Δ*T*_*m*_ < 0). The color code refers to significant proteins by RTSA (see panel **A**). GRASP55 is the RTSA significant protein with the highest statistical significance among the stabilized proteins and, thus, a highly promising prodigiosin target protein candidate. **C** Volcano-like plot of the statistical significance vs. the extent of prodigiosin-mediated change in protein abundance (higher or lower for mean log_2_ ratio > 0 or < 0, respectively) calculated from protein intensities at 36.5 °C. The color code refers to significant proteins by RTSA (see panel **A**). **D** Melting curves (solid lines) of GRASP55 from prodigiosin or DMSO treated cells (RTSA software output of TPP-TR analysis). Datapoints and whiskers represent the arithmetic mean ± SD of three replicates. Datapoints for temperatures showing significant intensity differences are dash-boxed, next to which the collation ratio and (uncorrected) p-value are given. The thermal stabilization of GRASP55 by prodigiosin (with a mean melting point difference of 2.1 °C) is indicated by dashed lines and a measure for statistical significance is provided (-lg(p), same as y-axis of panel **B**). **E** Immunoblotting for GRASP55 protein quantification from the non-denatured protein fractions of prodigiosin or DMSO treated HeLa cells (CETSA). **F** Melting curves of GRASP55 from quantitative immunoblotting (CETSA, see panel **E**) using the same RTSA analysis and representation as for TPP-TR (see panel **D**). The thermal stabilization of GRASP55 by prodigiosin was confirmed

For prodigiosin-treated (100 nM, 6 h) HeLa cells, prodigiosin-affected differential melting (recorded at ten distinct temperature points in the range between 36.5 °C – 67 °C) and abundance data were obtained for 2480 proteins selected according to the data quality criteria of the RTSA software [33] (Supplementary Table S1). Among the prodigiosin-affected proteins (38 with positive and 55 with negative RTSA distance score) determined by the RTSA analysis (Fig. 3A; combined thermal stability and abundance effect), GRASP55 was the statistically most significant thermally stabilized protein (Fig. 3B), suggesting a direct (or indirect) prodigiosin interaction. No effect of prodigiosin on GRASP55 abundance was observed (Fig. 3C), indicating that prodigiosin does not affect GRASP55 expression levels (at 100 nM within 6 h). Melting curves for GRASP55 showing these prodigiosin effects (thermal stabilization without abundance alternation) are given in Fig. 3D. As described for the CETSA assay [29], we also quantified the non-denatured fraction of GRASP55 by immunoblotting (Fig. 3E and F) and, thus, validated and verified the prodigiosin-induced stabilization of GRASP55 as detected by MS-based TPP-TR.

A STRING protein–protein association network analysis [66] of the 93 prodigiosin-affected proteins revealed several clusters including fatty acid metabolism, lysosome, and autophagy associated proteins (Fig. S2). Moreover, 22 of these 93 proteins are associated with the Golgi apparatus (GO:0005794), thereof twelve with the Golgi membrane (GO:0000139), and thereof six with Golgi-associated vesicles (GO:0005798). Considering these secondary prodigiosin-mediated effects, the Golgi protein GRASP55, although not a central node in the protein network, represents a highly interesting potential direct (or indirect) molecular target candidate of prodigiosin, as it has been previously described to be involved in unconventional secretion [1, 53] and in physically linking autophagosomes with lysosomes [80].

### GRASP55 is stabilized at low nanomolar prodigiosin concentrations

**Fig. 4:**
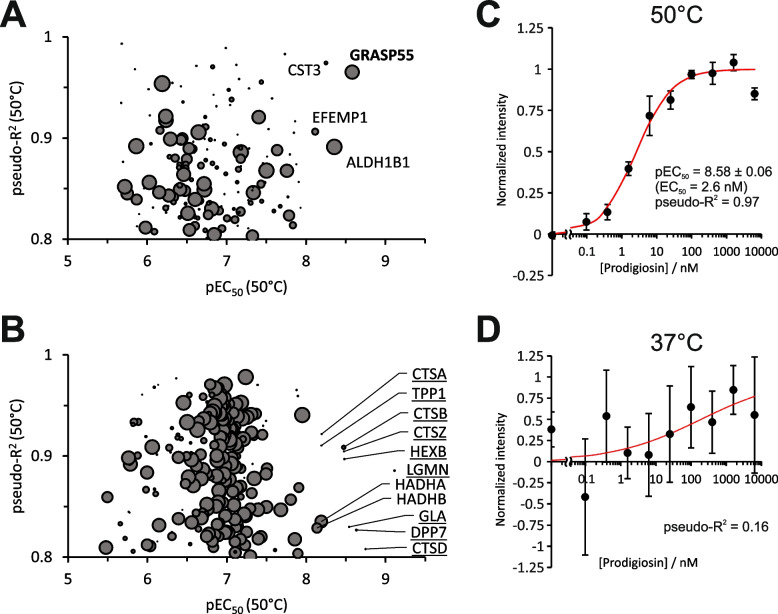
GRASP55 is stabilized at low nanomolar prodigiosin concentrations. For thermal proteome profiling compound concentration range (TPP-CCR) experiments, HeLa wt cells were treated with ten different concentrations of prodigiosin for 6 h, harvested, and cell suspensions were exposed to a short (3 min) constant temperature treatment at 50 °C (or 37 °C to test for abundance effects). Cells were lysed and the non-denatured protein fraction was recovered after centrifugation followed by quantitative MS analysis as described for TPP-TR, resulting in dose response characteristics for prodigiosin-affected proteins. **A** Plot of the dose response curve fitting parameters pseudo-R^2^ (proteins with a pseudo-R^2^ value of > 0.8 were considered to have prodigiosin dose–response characteristics) vs. pEC_50_ (the negative decadic logarithm of the half-maximal effective concentration) for proteins exhibiting increasing intensities at increasing prodigiosin concentrations (positive dose response). The data point diameter encodes the pseudo-R^2^ (50 °C – 37 °C) difference (high for solely (de)stabilized proteins, low for solely abundance affected proteins). Proteins with pEC_50_ > 8 (EC_50_ < 10 nM) are labeled. GRASP55 is the protein affected (stabilized) at the lowest prodigiosin concentrations among the proteins with positive dose response. **B** Same representation as for panel **A** but for proteins with negative dose response. Lysosome associated proteins (UniProt annotated keyword KW-0458) are underlined. **C** Dose response characteristics and fitting results for GRASP55 at 50 °C. Data points and whiskers represent the arithmetic mean ± SD of three replicates and the fitted dose response curve is shown in red. **D** Same representation as in panel **C** but for 37 °C, with the absence of a dose–response effect (irrelevant pseudo-R2 <  < 0.8) represented by a thin dotted fit curve (in red)

To investigate the affinity of prodigiosin to potential protein targets, we estimated the prodigiosin concentration at which 50% of the stabilizing effect can be observed (EC_50_: half-maximal effective concentration, pEC_50_: negative decadic logarithm of EC_50_ on the molar scale) by TPP compound concentration range (TPP-CCR) [62]. In contrast to TPP-TR performed at different denaturation temperatures and fixed prodigiosin concentrations, for TPP-CCR, HeLa cells were treated with different concentrations of prodigiosin for 6 h and subsequently heated for 3 min at a fixed temperature of 50 °C, around which most proteins exhibit partial melting and, if present, small molecule induced effects on melting characteristics [62]. In line with the TPP-TR results, the vast majority of proteins (3741 of 4139 proteins with at least two replicates of full dose response data excluding contaminants, reverse hits and only by site identifications; Supplementary Table S2) remained unaffected by prodigiosin treatment in these TPP-CCR experiments, rated by a pseudo-coefficient of determination (pseudo-R^2^; the term “pseudo” refers to employing a non-linear model) below 0.8 of the sigmoidal dose response curve fit. With pseudo-R^2^ ≥ 0.8, 162 proteins exhibited increasing (Fig. 4A) and 236 proteins decreasing (Fig. 4B) dose response characteristics. To distinguish thermal (de)stabilization from abundance effects, a control data set at 37 °C was generated. In case of pure thermal stability alteration, the absence of dose response effects is expected at 37 °C, whereas an abundance effect would lead to similar dose response characteristics at 37 °C as for 50 °C and, thus, to similar pseudo-R^2^ values at both temperatures, i.e., a low pseudo-R^2^ (50 °C – 37 °C) difference (encoded by the point size in Fig. 4A and B). Of the 162 proteins with increasing dose response characteristics at 50 °C (Fig. 4A), GRASP55 was affected at the lowest prodigiosin concentration (pEC_50_ = 8.58 ± 0.06, EC_50_ = 2.6 nM, Fig. 4C) with a very high pseudo-R^2^ (0.97), whereas, for samples treated at 37 °C, no dose response effects were observed for GRASP55 (Fig. 4D). We conclude that GRASP55 is stabilized at low nanomolar prodigiosin concentrations at a steady abundance level, suggesting, together with the effects on Golgi apparatus structure, GRASP55 as a highly probable direct (or indirect) target of prodigiosin.

Of the proteins exhibiting dose response characteristics at 50 °C with negative slope, i.e., decreasing intensity (Fig. 4B), the nine proteins with the highest pEC_50_ (underlined in Fig. 4B) are all associated with the lysosome (UniProt annotated keyword KW-0458) and exhibit similar pseudo-R^2^ values for 50 °C and 37 °C (low pseudo-R^2^ (50 °C – 37 °C) difference expressed by small datapoints in Fig. 4B), indicating a decrease in abundance, e.g., by downregulation, degradation or secretion. This result is in line with the abundance decrease of the similar lysosomal protein cluster observed for the TPP-TR experiments (Fig. 3C and S2). The other two proteins with pEC_50_ > 8 (HADHA/B) were mainly thermally destabilized (relatively high pseudo-R^2^ (50 °C – 37 °C) difference) in accordance with the TPP-TR results, where they grouped in the fatty acid metabolism protein cluster (Fig. S2).

### Knockout of GRASP55 inhibits prodigiosin cytotoxicity and alters prodigiosin effects on the Golgi apparatus

**Fig. 5:**
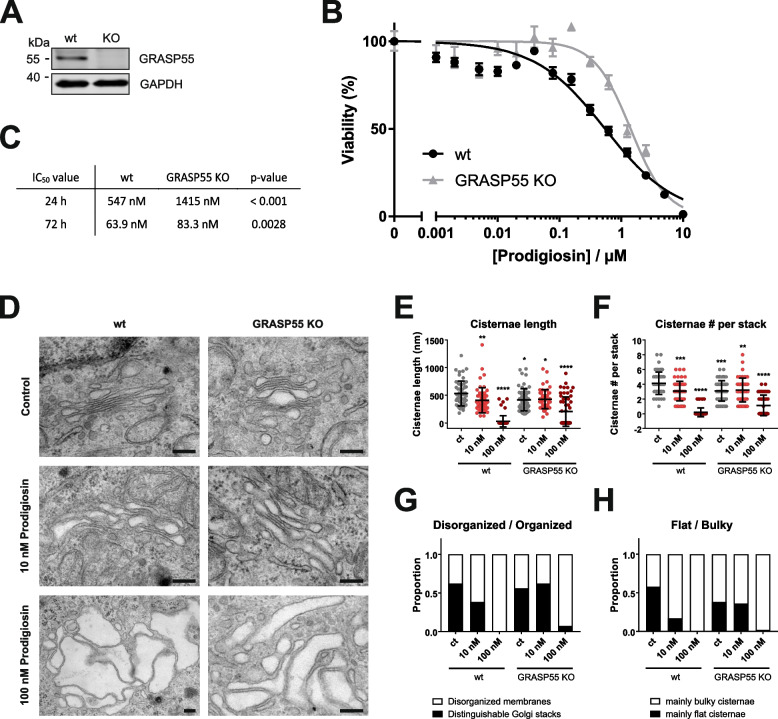
Knockout of GRASP55 impairs prodigiosin cytotoxicity and alters prodigiosin effects on the Golgi apparatus. **A** GRASP55 was knocked out in HeLa wt cells and KO was verified by western blot. **B** HeLa wt and HeLa GRASP55 KO cells were treated with different concentrations of prodigiosin for 24 h. After treatment, cell viability was measured using a thiazolylblue (MTT) assay. Results are shown as the mean ± SEM of three independent experiments performed in triplicates for each treatment. **C** IC_50_ values and statistical analysis for MTT assays in HeLa wt and HeLa GRASP55 KO cells after 24 and 72 h treatment with prodigiosin. Please note that the MTT assay for HeLa wt cells was independent from the one shown in Fig. 1B; accordingly, IC_50_ values slightly differ. (**D-H**) HeLa wt and HeLa GRASP55 KO cells were treated with DMSO, 10 nM or 100 nM prodigiosin for 24 h and effects on Golgi apparatus structure were investigated by transmission electron microscopy. **D** Representative electron micrographs are shown. Scale bar: 200 nm. **E** Cisternae length and **F** cisternae number per stack were quantified. **G** Golgi stacks were categorized into organized (stacked structures with three or more cisternae) and disorganized (multiple unstacked cisternae and vesicles). **H** Stacks were classified as containing mainly flat or mainly bulky/swollen cisternae. At least 50 Golgis per treatment were quantified after blinding and randomization. Bars represent the means ± SD. p values were determined by ordinary one-way ANOVA with Dunnett´s post hoc test. * *p* < 0.05; ** *p* < 0.01; *** *p* < 0.001; **** *p* < 0.0001

The TPP results and the results of the ultrastructural analysis of the Golgi apparatus encouraged us to investigate prodigiosin cytotoxicity in cells deficient for GRASP55. For that purpose, we generated a GRASP55 KO HeLa cell line as described by Bekier et al. (Fig. 5A) [5]. In GRASP55 KO cells, prodigiosin still displayed a high cytotoxicity (Fig. 5B), but IC_50_ values were significantly increased in comparison to HeLa wild-type (wt) cells after both 24 and 72 h (Fig. 5C). After 24 h treatment with prodigiosin, the IC_50_ value of KO cells is approximately threefold higher than in wt cells. These results suggest that cytotoxic effects of prodigiosin partially depend on GRASP55 and/or its cellular function.

Utilizing TEM, severe changes in Golgi apparatus morphology after treatment with prodigiosin became apparent in HeLa wt cells (see Fig. 1B-D). Since GRASP55 is a major protein for the stacking of the *trans* Golgi cisternae, we decided to investigate the influence of prodigiosin treatment on Golgi apparatus structure in GRASP55 KO cells (Fig. 5D). It has been shown previously that deletion of GRASP55 leads to an impairment of the Golgi stacking, as displayed in partly swollen and shorter Golgi cisternae and a higher frequency of disorganized Golgi membranes [5]. Both prodigiosin treatment and GRASP55 KO resulted in a reduced cisternae length (Fig. 5E) and cisternae number per Golgi stack (Fig. 5F), but effects of GRASP55 KO and prodigiosin treatment were not additive. Quantification of the TEM images revealed that the ratio between disorganized membranes and distinguishable Golgi stacks was clearly increased after treatment with 10 nM prodigiosin compared to the DMSO control in HeLa wt cells (Fig. 5G). In contrast, no change in the ratio between disorganized membranes and distinguishable Golgi stacks was observed in HeLa GRASP55 KO cells after treatment with 10 nM prodigiosin. Severe structural changes of the Golgi apparatus could be observed in both wt and KO cells after 24 h treatment with 100 nM prodigiosin. No well-organized Golgi could be observed in the perinuclear region, instead we found groups of vacuoles and irregularly dilated non-stacked cisternae. In addition, we classified Golgi structures into containing predominantly flat or bulky/swollen cisternae (Fig. 5H). Treatment with 10 nM prodigiosin reduced the amount of mainly flat cisternae in wt cells, but not in GRASP55 KO, whereas treatment with 100 nM prodigiosin resulted cell line-independently in heavily dilated Golgi structures.

Since alterations in Golgi apparatus morphology have been associated with altered protein secretion [77], we investigated cellular secretion in HeLa wt and HeLa GRASP55 KO cells after treatment with 100 nM prodigiosin for 24 h (Fig. S3). Beside profound changes in the pattern of secreted proteins induced by GRASP55 knockout, we found also prodigiosin-mediated abundance changes of certain protein groups differing between wt and KO cells. Notably, proteins containing signal peptides (SP)—which might represent classically secreted proteins—showed higher abundances in the secretome of GRASP55 KO cells upon prodigiosin treatment which was not apparent in wt cells. Furthermore, proteins associated with the extracellular matrix (ECM) and the lysosome were higher abundant after prodigiosin treatment exclusively in KO cells. Taken together, these results support the hypothesis that the Golgi stacking protein GRASP55 represents a protein target of prodigiosin.

### Prodigiosin treatment leads to the accumulation of autophagosomes at the Golgi apparatus and blocks autophagic flux

**Fig. 6:**
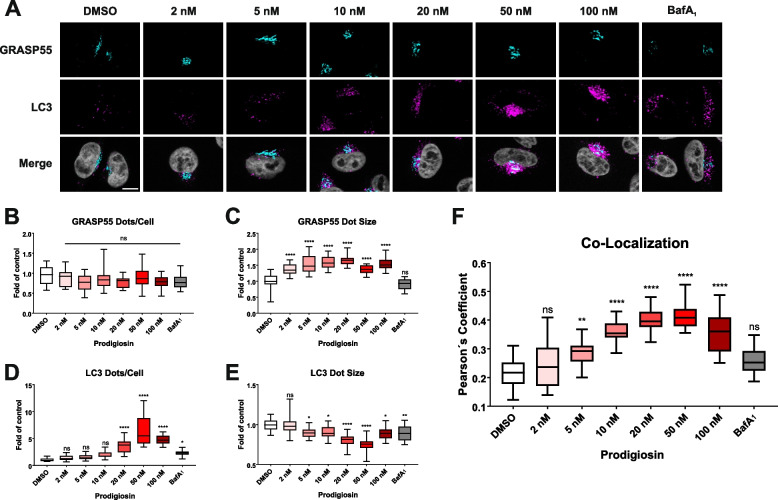
GRASP55 and LC3 co-localize upon treatment with prodigiosin. HeLa wt cells were seeded on cover slips. On the next day, cells were treated with different concentrations of prodigiosin, 10 nM bafilomycin A_1_ (BafA_1_) or DMSO for 6 h. After treatment, cover slips were prepared for microscopy. **A** Representative sections are depicted. Scale bar: 10 µm. **B-E** The relative number per cell and mean area of GRASP55 and LC3 positive structures and **F** the co-localization (Pearson´s coefficient) after Costes thresholding of GRASP55 and LC3 of 15 representative images from three biological replicates for each treatment were quantified using ImageJ 1.53c. p values were determined by ordinary one-way ANOVA with Dunnett´s post hoc test. * *p* < 0.05; ** *p* < 0.01; *** *p* < 0.001; **** *p* < 0.0001; ns, non-significant

It has been previously reported that GRASP55 participates in the autophagic process by promoting autophagosome-lysosome fusion through binding to the autophagosome-bound ubiquitin-like protein microtubule-associated proteins 1A/1B light chain 3 ([MAP1]LC3; LC3 hereafter) [81]. Since modulation of autophagy has been reported as one mechanism of action of prodigiosin and its derivatives by others and us [6, 10, 35, 36, 82], our next aim was to investigate if prodigiosin modulates the interplay between GRASP55 and autophagy by analyzing the subcellular localization of GRASP55 and LC3 upon treatment with different concentrations of prodigiosin or the late-stage autophagy inhibitor bafilomycin A_1_ (BafA_1_) as a control (Fig. 6A). After all applied treatments, GRASP55 was observed in the perinuclear region, which is consistent with the localization of the Golgi apparatus in literature [77]. The number of GRASP55 dots/cell remained unaltered upon different treatments (Fig. 6B). Interestingly, after treatment with all concentrations of prodigiosin, but not BafA_1_, the mean area of GRASP55 dots was significantly increased compared to the DMSO control (Fig. 6C). These results are in line with the observation of an enlarged Golgi in TEM and BFA washout assays. LC3-positive structures can be observed as dots, which represent autophagosomes. As expected, LC3 dots were enriched after treatment with the autophagy inhibitor BafA_1_ (Fig. 6D). In prodigiosin treated cells, the number of LC3 dots increased dose-dependently and the number of LC3 dots was higher than after treatment with BafA_1_ (Fig. 6D). Furthermore, after both treatments, LC3-positive structures are significantly smaller than in DMSO treated cells (Fig. 6E). We also analyzed the co-localization of LC3 and GRASP55 and observed a highly significant, dose-dependent increase in co-localization of these two proteins after treatment with prodigiosin, whereas after treatment with BafA_1_ there is no significant increase in co-localization (Fig. 6F). This observation suggests an accumulation of autophagosomes or their precursor membranes at the Golgi apparatus or in its close vicinity.

**Fig. 7:**
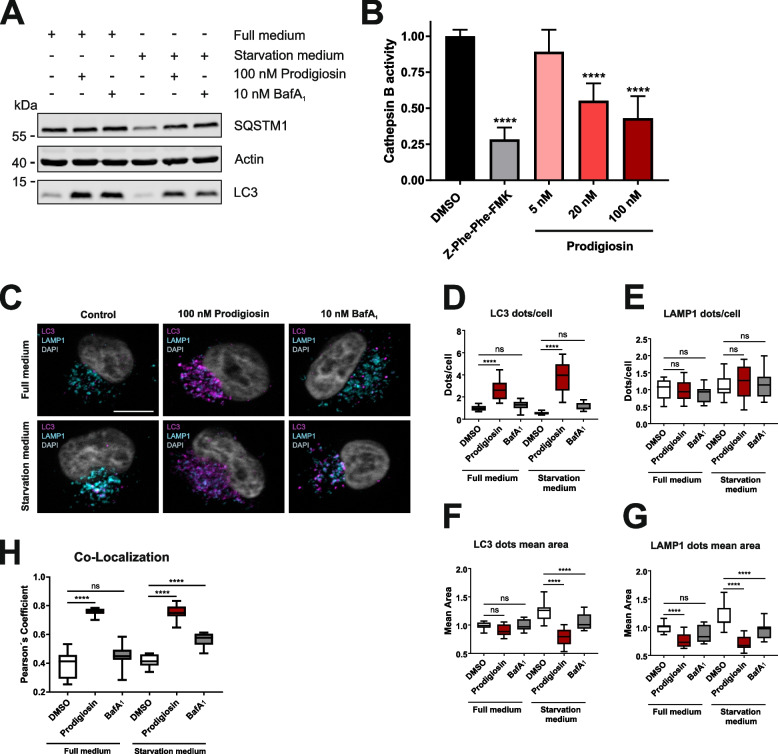
Prodigiosin blocks autophagy and inhibits cathepsin activity. **A** HeLa wt cells were treated with DMSO, 100 nM prodigiosin or 10 nM bafilomycin A_1_ (BafA_1_) in DMEM or EBSS. After 6 h, the cells were lysed and cellular lysates were immunoblotted for the indicated proteins. One representative immunoblot of three independent experiments is shown. LC3: light chain 3; SQSTM1: sequestosome 1. **B** HeLa wt cells were treated with different concentrations of prodigiosin or DMSO. After 24 h, the cells were lysed and a cathepsin B assay was performed according to the manufacturer´s instructions. 20 µM Z-Phe-Phe-FMK was used as inhibitor control. The fluorescence of duplicates for each treatment of three independent experiments was measured and the mean of the DMSO control was set as 100%. Bars represent the means + SD. **C** HeLa wt cells were seeded on cover slips. On the next day, cells were treated with DMSO, 100 nM prodigiosin or 10 nM BafA_1_ in DMEM or EBSS for 2 h. After treatment, cover slips were prepared for microscopy. Representative sections are depicted. Scale bar: 10 µm. **D-G** The relative number per cell and mean area of LC3- and LAMP1-positive structures and **H** the co-localization (Pearson´s coefficient) after Costes thresholding of LC3 and LAMP1 of 15 representative images from three biological replicates for each treatment were quantified using ImageJ 1.53c. p values were determined by ordinary one-way ANOVA with Dunnett´s post hoc test. **** *p* < 0.0001; ns, non-significant

To investigate effects on the autophagic flux directly, immunoblot analysis of LC3 and of the ubiquitin-binding protein p62 (also known as sequestosome 1 [SQSTM1]) often represent the method of choice. Increased levels of the autophagosome-bound, lipidated form of LC3 (termed LC3-II) occur when autophagy is induced but also when the autophagic process is blocked in later steps. Investigation of the autophagy receptor SQSTM1, which accumulates upon autophagy blocking, can help distinguish between autophagy induction and inhibition. After 6 h of treatment with prodigiosin, LC3-II accumulated and the starvation-induced degradation of SQSTM1 was blocked in HeLa wt cells (Fig. 7A). These effects on the protein levels of LC3-II and SQSTM1 were similar to the effects of the V-ATPase inhibitor BafA_1_. BafA_1_ inhibits the acidification of lysosomes and thereby impairs the function of lysosomal proteases such as cathepsins and thus prevents the degradation of engulfed cargo and LC3-II [69]. We have previously described that the activity of pH-dependent cathepsins is severely reduced after treatment with prodigiosin in urothelial bladder carcinoma cells [6]. Likewise, in HeLa wt cells treatment with prodigiosin led to a significantly reduced cathepsin B activity in a concentration-dependent manner (Fig. 7B). To further investigate the effects of prodigiosin, we performed immunofluorescence analysis and stained for LC3 (autophagosomes) and lysosomal-associated membrane protein 1 (LAMP1) (Fig. 7C). In line with the immunoblot analysis, an increase of LC3-positive structures after treatment with prodigiosin was observed in immunofluorescence analysis (Fig. 7D). In contrast, unlike in immunoblot analysis, the increase of LC3-positive structures upon BafA_1_ treatment was only minor, which might be attributable to the shorter treatment duration. LAMP1-positive structures remained unaltered upon different treatments (Fig. 7E). Interestingly, both autophagosomes and lysosomes significantly shrank after treatment with prodigiosin, whereas the size difference to DMSO treated cells was not as prominent for BafA_1_ treated cells (Fig. 7F and G). Moreover, there was a highly significant increase in co-localization of LC3- and LAMP1-positive structures after treatment with prodigiosin, whilst changes after BafA_1_ treatment were comparatively low (Fig. 7H). The spatial proximity of autophagosomes and lysosomes after prodigiosin treatment would suggest a facilitated fusion of autophagosomes and lysosomes and therefore an increased autophagic flux. On the contrary, the inhibition of starvation-induced SQSTM1 degradation clearly indicates a blockage of autophagic flux. Collectively, these results suggest a different mechanism of autophagy inhibition by prodigiosin compared to BafA_1_. Prodigiosin treatment results in reduced cathepsin activity and blocks overall autophagic flux, whereas LC3-LAMP1 co-localization is clearly promoted. At the same time, the prodigiosin-induced accumulation of autophagosomes at GRASP55-positive structures points towards an involvement of an altered Golgi function in the autophagy-inhibitory effect of this natural compound.

## Discussion

Despite frequent advances in research, cancer remains a leading cause of death, accounting for nearly ten million deaths every year (https://www.who.int/news-room/fact-sheets/detail/cancer). To address this unmet therapeutic need, natural compounds display a valuable source for an almost inexhaustible diversity of bioactive compounds with a complexity in structure that is often superior to compounds derived from synthetic approaches. To elucidate the mechanism of action of these often highly bioactive compounds, TPP displays a valuable tool for unbiased target identification. In this study, we used TPP to identify the Golgi protein GRASP55 as target protein of the natural compound prodigiosin. We observed that prodigiosin treatment leads to severe structural changes in Golgi apparatus morphology and function. We also found that autophagosomes accumulate at the Golgi apparatus while autophagy is impaired in cells treated with prodigiosin.

Utilizing TPP, we identified several proteins as target candidates of the natural compound prodigiosin. By offering an unimpaired approach that is not dependent on pre-functionalization of the small molecule or of proteins, TPP represents a highly versatile target identification strategy [31]. Prioritizing candidates for target validation can be challenging due to multiple possible candidates. However, with severe structural changes in Golgi morphology, a high statistical significance of the thermal shift, validation through immunoblotting and being a target candidate in both independent TPP approaches (TPP-TR and TPP-CCR), GRASP55 was selected for further investigations. Distinguishing between thermal stability changes and abundance changes can be challenging [33], but neither in the TPP-TR/RTSA nor in the TPP-CCR analysis, GRASP55 showed differential intensities at 36.5/37 °C, excluding an abundance effect and establishing GRASP55 as a highly probable molecular target of prodigiosin with low nanomolar affinity.

To avoid focussing on false positive targets, Johnson et al. proposed orthogonal target identification methods like computational target prediction [31]. To our knowledge, only the N-terminal GRASP domain of GRASP55 (residues 1–207) has been successfully crystallized so far [41, 67], hindering reliable computational modelling of a direct prodigiosin-GRASP55 interaction. Future disclosure of the whole protein structure is desirable to find and characterize a potential specific binding site of prodigiosin. This knowledge would allow structure optimization of this highly active natural compound with regard to its binding affinity, pharmacokinetic properties and adverse effects. Of note, we found that KO of GRASP55 significantly reduces prodigiosin cytotoxicity in HeLa cells, indirectly confirming GRASP55 as a target. Xiang et al. reported that depletion of GRASP55 does not cause apoptosis in HeLa cells [77]. We do not think that prodigiosin-mediated effects on GRASP55 mimic its genetic depletion, but, next to apoptosis, additional cell death subroutines might be involved, e.g. lysosome-dependent cell death [23]. Prodigiosin is still clearly cytotoxic in GRASP55 KO cells, demonstrating that other target candidates and/or mechanisms contribute to the observed reduction in cell viability upon prodigiosin treatment. Along these lines, small molecules can be bioactive through mechanisms that do not involve direct binding of proteins and, thus, those target processes may not be revealed by TPP.

In our hands, prodigiosin treatment leads to a severely dilated and disorganized Golgi apparatus in a concentration-dependent manner. In mammalian cells, Golgi membranes are organized as stacks of multiple flat cisternae, which are further linked into a ribbon-like structure by the two peripheral membrane proteins GRASP55 and GRASP65 [37]. The conserved GRASP domain at the N-terminus of GRASP proteins contains a membrane anchor and forms dimers and trans-oligomers to glue the adjacent cisternae into stacks [2]. We speculate that low nanomolar concentrations of prodigiosin interfere with the oligomerization of GRASP55 leading to a Golgi phenotype with less and shorter Golgi cisternae that is similar to GRASP55 KO cells [5]. Notably, these effects become visible after treatment with 10 nM prodigiosin in EM analysis and GRASP55 immunofluorescence, but not after TGN46 and B4GALT1 immunostaining for BFA washout assays. Upon GRASP55 deletion, Bekier et al. observed minor but significant Golgi fragmentation by immunofluorescence [5]. Interestingly, after treatment with 100 nM prodigiosin, we observed heavily dilated and disorganized structures in both TEM and BFA washout assays. These morphological changes in Golgi morphology are similar to phenotypes observed in cells after treatment with the bacterial metabolite monensin [19, 49, 68]. As an ionophore, monensin is able to dissipate proton gradients by exchanging Na^+^/H^+^ across membranes leading to a disruption of the Golgi pH. Golgi apparatus function is dependent on a pH gradient that is maintained along the secretory pathway by proton pumps [15] with an acidic pH in the *trans*-cisternae and newly formed vesicles [3, 51, 54]. Several studies suggest that prodigiosin can alter organelle pHs by acting as an H^+^/Cl^−^ symporter [61, 63]. Therefore, we hypothesize that the alteration of Golgi apparatus morphology after treatment with prodigiosin is mediated by both direct effects on GRASP55 oligomerization and alkalization-induced cisternae swelling.

The Golgi apparatus is the central hub for post-translational modifications of proteins and their sorting and transport to their final destinations, such as secretory vesicles, endosomes, lysosomes, or the plasma membrane [7]. It has been hypothesized that accelerated protein trafficking and impaired protein glycosylation after unstacking of the Golgi cisternae due to GRASP55 depletion or impairment can be explained by an enlarged membrane area that is accessible for faster vesicle budding and cargo transport through the Golgi [70, 72]. Therefore, it is not surprising that in our hands the severe impairment of the Golgi apparatus structure led to alterations in the pattern of classically secreted proteins as well as proteins associated with extracellular matrix and lysosomes. This stands in line with the observations of Xiang et al. that Golgi unstacking after GRASP depletion led to a missorting of lysosomal enzymes such as cathepsin D [77]. Since GRASP55 is not only involved in conventional secretion due to its Golgi stacking activity, but also plays a role in unconventional protein secretion [24, 25, 53], it is comprehensible that we also found a prodigiosin mediated upregulation in the secretion of different protein groups. Taken together, we show that prodigiosin treatment leads to severe changes in Golgi morphology and function, which has—to our knowledge—not been described in the literature so far.

In addition to its functions in Golgi stacking and unconventional secretion, GRASP55 has been described as a specific energy and nutrient sensor [2] acting as a bridging protein that facilitates autophagosome-lysosome fusion through an LC3-interaction region (LIR) motif and interaction with LAMP2 [80, 81]. Prodigiosin and its autophagy-modulating properties have been discussed controversially in recent literature. While some groups describe an induction of autophagic cell death [10, 42], Zhao et al. report an inhibition of autophagy via blocking lysosomal cathepsin maturation and autophagosome-lysosome fusion [82]. In this study, we observed an increased spatial proximity of autophagosomes and lysosomes, as visualized by the co-immunostaining of LC3 and LAMP1 after treatment with prodigiosin. However, we also observed that prodigiosin ultimately blocks autophagic cargo degradation, as shown by the accumulation of LC3-II, the inhibition of SQSTM1 degradation, and a reduced cathepsin activity. The Golgi has previously been designated as “assembly line” to the autophagosome [14], and key regulators of the autophagic pathway traffic from the Golgi to the forming autophagosome, including the lipid scramblase ATG9A and the phosphatidylinositol 4-kinase PI4KIIIβ, which mediates PI4P production at the initiation membrane site [32]. Furthermore, the signalling of mTORC1—a major autophagy-regulating kinase—is closely linked to the morphology of the Golgi [44], and mTORC1 directly phosphorylates GRASP55 and thus regulates its localization [53]. Accordingly, a prodigiosin-mediated control of the autophagy pathway via its effects on Golgi structure in general and/or on GRASP55 in particular appears likely. Overall, we propose an at least dual mode of action how prodigiosin inhibits late stage autophagy. First, prodigiosin treatment leads to an alkalization of lysosomes by acting as an H^+^/Cl^−^ symporter [61, 63] and simultaneously impairs proper functionalization and trafficking of lysosomal proteins via unstacking of Golgi cisternae and/or alkalization of this compartment. Second, prodigiosin might directly interfere with the role of GRASP55 as a tethering factor for autophagosome-lysosome fusion. Further studies will be required to determine the individual contribution of these two possible modes of action. Notably, Zhang et al. reported that knockout of GRASP55 reduces LC3 and LAMP2 co-localization [80]. Since we observed a clear co-accumulation of LC3- and LAMP1-positive structures upon prodigiosin treatment, it appears that not necessarily the GRASP55-mediated tethering of autophagosomes and lysosomes itself is negatively affected by this compound. Alternatively, it might be that the prodigiosin-mediated Golgi unstacking or pH alterations influence biogenesis and trafficking of LC3- and/or LAMP1-positive compartments. Along these lines, we also observed reduced sizes of these puncta, potentially pointing towards an altered transfer of proteins and/or lipids into these structures. The general importance of the Golgi apparatus for autophagosome biogenesis during starvation has been described above. Due to the severe damage of the Golgi inflicted by prodigiosin, selective autophagy processes (i.e., Golgiphagy) might add another layer of complexity in this case. The massive co-localization of GRASP55 and LC3 induced by prodigiosin might reflect the de novo synthesis of autophagosomal membranes at the damaged organelle. The induction of Golgiphagy together with the block of late stage autophagy might explain the extreme accumulation of autophagosomal membranes in the perinuclear area upon prodigiosin treatment.

In 2013, Krishna et al. published an in silico molecular docking analysis of prodigiosin and cycloprodigiosin as COX-2 inhibitors [38]. Additionally, the identification of protein targets of prodigiosin using inverse virtual screening methods has been described in a recent manuscript, and reported potential prodigiosin-interacting proteins include HER-2, MEK, and S6K [55]. However, the interaction of prodigiosin with these candidate proteins has not been experimentally proven so far. Here, we identify GRASP55 as downstream effector protein of prodigiosin and characterize the immense effects of this bacterial metabolite on Golgi apparatus structure and function. Increased knowledge about the protein target enables the structural modification of this very potent natural compound in order to obtain a more selective mode of action and optimize the binding affinity and pharmacokinetic properties. The sensitivity of cancer cells towards prodigiosin treatment and the concomitant absence of effects on normal or non-malignant cells has previously been proposed [26, 50], making this natural product a promising lead compound for anti-cancer drug discovery. We hope that our results contribute to deciphering the molecular mode of action of this compound.

## Material and methods

### Antibodies and reagents

Antibodies against β-actin (Sigma-Aldrich, St. Louis, MO, USA, #A5316, clone AC-74, 1:5000), B4GALT1 (Sigma-Aldrich, St. Louis, MO, USA, #HPA010807, 1:200), GAPDH (abcam, Cambridge, UK, #ab8245, 1:5000), GRASP55 (Proteintech, Chicago, IL, USA, #10,598–1-AP, 1:200 for IF and 1:2000 for WB), LAMP1 (Sigma-Aldrich, St. Louis, MO, USA, #L1418, 1:200), LC3B (MBL, Woburn, MA, USA, #M-152–3, 1:200 for IF and Cell Signalling Technology, Danvers, MA, USA, #2775, 1:1000 for WB), SQSTM1 (PROGEN, Heidelberg, Germany, #GP62-C, 1:1000) and TGN46 (Bio-Rad, Hercules, CA, USA, #AHP500GT, 1:200) were used. For WB, IRDye 800- or IRDye 680-conjugated secondary antibodies were purchased from LI-COR Biosciences (Lincoln, NE, USA, #926–68,077, #926–32,211 and #926–32,210). Secondary antibodies for immunofluorescence analyses were purchased from Jackson ImmunoResearch (Alexa Fluor 488-AffiniPure Goat Anti-Mouse IgG, 1:500, #115–545-003; Alexa Fluor 488-AffiniPure Goat Anti-Rabbit IgG, 1:500, #111–545-003; Alexa Fluor 647-AffiniPure Goat Anti-Mouse IgG, 1:500, #115–605-003 and Alexa Fluor 647-AffiniPure Goat Anti-Rabbit IgG, 1:500, #111–605-144) and abcam (Alexa Fluor 488 Donkey Anti-Sheep IgG, 1:500, #ab150177). Isolated and purified prodigiosin was provided in DMSO as reported previously [20]. Other reagents used were bafilomycin A_1_ (Sigma-Aldrich, St. Louis, MO, USA, #B1793), brefeldin A (BFA, Sigma-Aldrich, St. Louis, MO, USA, #B6542), DMSO (PanReac AppliChem, Darmstadt, Germany, #A3672 and ROTH, Karlsruhe, Germany, #7029.1), staurosporine (biomol, Hamburg, Germany, #AG-CN2-0022-M005), Thiazolyl blue (MTT, ROTH, Karlsruhe, Germany, #4022.3) and Z-Phe-Phe-FMK (abcam, Cambridge, UK, #ab65306). The cathepsin activity of treated HeLa cells was quantified using the fluorimetric Cathepsin B Activity Assay Kit (abcam, Cambridge, UK, #ab65300) according to the manufacturer´s instructions and measured with a microplate reader (SynergyMx, BioTek, Winooski, VT, USA).

### Correct Identification of natural products

Prodigiosin was produced and purified as described by Domröse et al. [20]. After column chromatography prodigiosin was precipitated as hydrochloride as a dark red solid and a 10 mM stock in DMSO was prepared.

^**1**^**H-NMR** (600 MHz, CDCl_3_): δ [ppm] = 0.90 (t, ^3^*J*_10″,9″_ = 7.0 Hz, 3H, 10″-H), 1.32 (m_c_, 4H, 8″-, 9″-H), 1.54 (m_c_, 2H, 7″-H), 2.39 (t, ^3^*J*_6″,7″_ = 7.6 Hz, 2H, 6″-H), 2.54 (s, 3H, 11″-H), 4.00 (s, 3H, 7-H), 6.07 (d, ^4^*J*_3,1_ = 1.9 Hz, 1H, 3-H), 6.35 (m_c_, 1H, 4′-H), 6.68 (d, ^4^*J*_3″,1″_ = 2.6 Hz, 1H, 3″-H), 6.91 (ddd, ^3^*J*_3′,4′_ = 3.8 Hz, ^4^*J*_3′,5′_ = 2.4 Hz, ^5^*J*_3′,1′_ = 1.4 Hz, 1H, 3′-H), 6.95 (s, 1H, 8-H), 7.22 (m_c_, 1H, 5′-H), 12.56 (brs, 1H, 1′-NH), 12.71 (brs, 2H, 1-, 1″-NH); ^**13**^**C-NMR** (151 MHz, CDCl_3_): δ [ppm] = 12.6 (C-11″), 14.2 (C-10″), 22.6 (C-9″), 25.5 (C-6″), 29.9 (C-7″), 31.6 (C-8″), 58.9 (C-7), 93.0 (C-3), 111.9 (C-4′), 116.1 (C-8), 117.2 (C-3′), 120.8 (C-5), 122.4 (C-2′), 125.3 (C-2″), 127.1 (C-5′), 128.5 (C-3″), 128.6 (C-4″), 147.1 (C-5″), 147.8 (C-2), 165.9 (C-4).

The analytical data are in accordance to literature [20].

### Cell lines and cell culture

All HeLa cell lines were cultured in Dulbecco´s Modified Eagle Medium (DMEM, Thermo Fisher Scientific, Waltham, MA, USA, #41,965,039) containing 10% Fetal Bovine Serum (FBS, Sigma-Aldrich, St. Louis, MO, USA, #F0804), 4.5 g/l D-glucose, 100 units/mL penicillin and 100 µg/mL streptomycin (Thermo Fisher Scientific, Waltham, MA, USA, #15,140,122). All cells were cultivated and treated at 37 °C and 5% CO_2_ in a humidified atmosphere.

### Cell viability assay

Cell viability was measured using the MTT (3-(4,5-dimethylthiazol-2-yl)-2,5-diphenyltetrazolium bromide) assay. HeLa cells were seeded in 96-well plates with a density of 5*10^3^ cells/well. One day after seeding, cells were treated with different concentrations of prodigiosin, 0.1% DMSO as a solvent control or 5 µM staurosporine as a positive control for 24 or 72 h. After the incubation time, 20 µL of a 5 mg/mL MTT stock solution (ROTH, Karlsruhe, Germany, #4022.3) were added to the cells and they were incubated at 37 °C and 5% CO_2_ in a humidified atmosphere for 30 min. Upon removal of the MTT-containing medium 100 µL DMSO per well were added for extraction of the formazan. Absorbance was measured at 570 nm and 650 nm (reference) with a microplate reader (SynergyMx, BioTek, Winooski, VT, USA). After subtraction of the reference value, the mean of the absorbance of the solvent control was set as 100% and the relative viability was calculated for each sample.

### Transmission electron microscopy (TEM)

HeLa wt and HeLa GRASP55 KO cells were seeded into 10 cm dishes. On the next day, cells were treated with 0.1% DMSO, 10 nM prodigiosin or 100 nM prodigiosin for 24 h. After treatment, cells were washed with Dulbecco´s phosphate-buffered saline (DPBS, Thermo Fisher Scientific, Waltham, MA, USA, #14,190,250) and then fixed using 2.5% glutaraldehyde and 4% paraformaldehyde in 0.1 M sodium cacodylate, pH 7.4 overnight at 4 °C. Then, cells were harvested with a cell scraper and centrifuged at 4,000 × *g* for 5 min. Cell pellets were washed twice with 0.1 M sodium cacodylate buffer, pH 7.2, then heated to 40 °C and embedded into 3% low melting agarose. The agarose was dissolved at 40 °C in a water bath and after aspiration of the supernatant a volume of approximately 10 µL was left, which was resuspended in agarose. After centrifugation at approximately 4,000 x *g* for 2 min, the samples were covered with 1% OsO_4_ in 0.1 M sodium cacodylate buffer for 60 min at RT. After washing two times with sodium cacodylate buffer for 10 min and twice with 70% EtOH for 15 min at RT, block contrast was applied using 1% uranyl acetate/1% phosphorotungstic acid in 70% EtOH (freshly prepared and filtered) for 1 h at RT. The samples were dehydrated in a graded ethanol series (90% EtOH, 96% EtOH, 100% EtOH) and embedded in SPURR epoxy resin (Serva, Heidelberg, Germany, #21,050). After polymerization at 70 °C for 24 h, 70 nm ultrathin sections were cut using an Ultracut EM UC7 (Leica, Wetzlar, Germany). TEM images were captured using an H7100 TEM (Hitachi, Tokyo, Japan) at 100 V equipped with a Morada camera (EMSIS GmbH, Münster, Germany).

### BFA washout assay

For BFA washout assays, HeLa wt cells were seeded on glass coverslips in 24-well plates. On the next day, cells were treated with 0.1% DMSO, 10 nM prodigiosin or 100 nM prodigiosin. After 24 h, the treatment medium was removed, cells were washed once with DPBS and then treated with 5 µg/mL BFA or 0.1% DMSO as a control for 2 h. Cells were then washed with DPBS four times and incubated with fresh culture medium for 0/15/30/45/60/120 min. After the respective BFA washout time, cells were fixed in ice-cold methanol for 15 min on ice, washed three times with DPBS and blocked in 3% BSA (Roth, Karlsruhe, Germany, #8076) overnight. Samples were incubated with primary antibodies diluted in 3% BSA for 2 h and then washed three times with DPBS, incubated with the appropriate secondary antibodies diluted in 3% BSA for 30 min and washed three times with DPBS. Afterwards, cells were embedded in ProLong Glass Antifade Mountant (Thermo Fisher Scientific, Waltham, MA, USA, #P36980) containing 1 µg/mL DAPI (Roth, Karlsruhe, Germany, #6335.1). Images were recorded with an Axio Observer 7 fluorescence microscope (Carl Zeiss Microscopy, Oberkochen, Germany) using a 40x/1,4 Oil DIC M27 Plan-Apochromat objective (Carl Zeiss Microscopy, Oberkochen, Germany) and an ApoTome 2 (Carl Zeiss Microscopy, Oberkochen, Germany).

### Thermal proteome profiling

TPP was performed essentially as described [40, 65], but with major modifications to the TMT labeling sets and statistical data analysis for TPP-TR.

#### Compound and temperature treatment

For thermal proteome profiling temperature range (TPP-TR) experiments, 6*10^6^ HeLa wt cells were seeded per 15 cm dish and, on the next day, incubated with 100 nM prodigiosin (final concentration in final 0.1% v/v DMSO in cell culture medium) or 0.1% v/v DMSO in cell culture medium as vehicle control for 6 h. For thermal proteome profiling compound concentration range (TPP-CCR) experiments, 2.35*10^6^ HeLa wt cells were seeded per 10 cm dish and, on the next day, incubated with the indicated final concentrations (Table 1) of prodigiosin (in final 0.1% v/v DMSO in cell culture medium) for 6 h.

**Table 1:** TPP-CCR treatment concentrations

Sample	1	2	3	4	5	6	7	8	9	10
[Prodigiosin] / nM	0	0.098	0.39	1.56	6.25	25	100	400	1600	6400

Adherent cells were washed in the dish once with DPBS and harvested by Trypsin–EDTA (0.25%, Thermo Fisher Scientific, Waltham, MA, USA, #25,200,056) treatment. After addition of 35 mL DPBS, the cells were pelleted by centrifugation (300 rcf, 5 min, 4 °C), and washed twice by re-suspending and pelleting using 45 mL and 1 mL DPBS, respectively, to remove excess trypsin and for transfer into pre-weighed 2 mL Eppendorf tubes to determine the cell pellet wet weights as quality check after centrifugation (300 rcf, 5 min, 4 °C) and complete removal of the supernatant. Cells for TPP-TR were resuspended using 1 mL, and cells for TPP-CCR were resuspended using 0.4 mL ice-cold DPBS, respectively, supplemented with protease inhibitor cocktail (Roche, Basel, Switzerland, #5,892,791,001). TPP-TR samples were aliquoted into 10 × 100 µL, and 2 × 100 µL aliquots were generated from TPP-CCR samples, respectively, into PCR tube strips such that samples with different prodigiosin concentrations and the same treatment temperature (described below) were contained in the same strip. Cell suspensions were shortly centrifuged (1 s or less) using a benchtop centrifuge to release trapped air and to achieve an even liquid level without pelleting the cells. The samples were then temperature treated by a 7 min pre-incubation at RT, 3 min temperature treatment in the PCR cycler (DNA Engine Tetrad 2, lid temperature 70 °C) at different temperatures (37 °C or 50 °C for TPP-CCR or the temperatures given in Table 2 for TPP-TR) and a 3 min post-incubation at RT in a metal heating block for uniform heat dissipation.

**Table 2:** TPP-TR treatment temperatures

PCR strip	1	2	3	4	5	6	7	8	9	10
Treatment temperature / °C	36.5	41.2	44.0	47.1	49.8	53.3	56.0	59.2	64.0	67.0

After temperature treatment, samples were supplemented with lysis buffer (final concentrations: 0.36 U/µL benzonase, 1.5 mM MgCl_2_, 1 mM Na_3_VO_4_, 10 mM NaF, 2.5 mM Na_4_P_2_O_7_, 0.8% w/v NP-40) for 1 h on ice. The lysates were cleared from cell debris as well as denatured and precipitated proteins by centrifugation at 20,000 rcf and 4 °C for 30 min. The total protein concentration of the resulting cell extracts containing the fraction of soluble, non-denatured proteins was determined (Pierce 660 nm Protein Assay, BSA as standard). Cell extracts were shock frozen in liquid nitrogen and stored at -80 °C. The three replicates for TPP-TR and TPP-CCR were prepared on consecutive days, respectively. The two (37 °C or 50 °C) x ten (concentrations) x three (replicates) = 60 TPP-CCR samples and two (treatment and control) x ten (temperatures) x three (replicates) = 60 TPP-TR samples were analyzed by LC–MS/MS. GRASP55 was additionally quantified from the TPP-TR samples by immunoblotting as described in the respective section.

#### Single-pot, solid-phase-enhanced sample preparation (SP3) for MS

For TPP-CCR, SP3 was performed as described [40] with slight modifications to the original protocol [28] using on average 5 µg total protein per sample and resulting in theoretically 5 µg peptides in 22 µL 50 mM triethylammonium bicarbonate per sample. For each of the two (treatment and control) x three (replicates) = six TPP-TR temperature treatment sample sets, the same volume for each of the ten temperature samples was used for SP3 processing, thus, maintaining the information about the temperature dependent non-denatured protein content. This volume was calculated such that the lowest two temperature samples (36.5 °C and 41.2 °C) contained 10 µg total protein on average. The samples were diluted using SDS containing buffer (20 µL final, final concentrations: 7.5% glycerol, 3% SDS, 37.5 mM Tris/HCl pH 7.0) and the proteins were reduced, alkylated, and precipitated on the solid phase as described [40] using adjusted volumes (2 × due to processing 10 µg instead of 5 µg). Volumes for washing of the aggregated proteins on the solid phase and for tryptic digestion were kept unchanged, however, to maintain the maximal total protein to trypsin/Lys-C ratio at 50:1 in the two rounds of digestion (13 h and 4 h), respectively, 2 × 0.2 µg trypsin/Lys-C was used per sample, theoretically resulting in at most about 10 µg peptides in 22 µL 50 mM triethylammonium bicarbonate.

#### TMT labeling and high pH fractionation

Peptides (10 µL of the peptide solutions, respectively, containing about 2.3 µg peptides for TPP-CCR or at most about 4.5 µg peptides for TPP-TR, respectively) were TMT labeled (1 µL for TPP-CCR or 2 µL for TPP-TR of the respective TMT 10plex label from 0.8 mg TMT label in 41 µL dry acetonitrile, 1 h, RT; quenched by 0.8 µl for TPP-CCR or 1.6 µL for TPP-TR of 2.5% w/v hydroxylamine, 15 min, RT), the samples of a labeling set were combined and offline high pH fractionated as described [40]. For TPP-CCR, a TMT 10plex labeling set contained the ten concentration samples according to the following scheme:

**Table:** 

[Prodigiosin] / nM	0	0.098	0.39	1.56	6.25	25	100	400	1600	6400
TMT label	126	127N	127C	128N	128C	129N	129C	130N	130C	131

For TPP-TR, however, in order to determine compound induced melting curve shifts and, at the same time, allow for precise relative protein quantification for differential protein expression analysis, corresponding temperature samples of treatment and control were kept within the same TMT 10plex labeling set and the ten different temperature treatments were split up into two TMT 10plex labeling sets according to the following scheme:

TMT 10plex labeling set 1

**Table:** 

Temperature / °C	64.0	64.0	56.0	56.0	49.8	49.8	44.0	44.0	36.5	36.5
Compound treatment	-	+	-	+	-	+	-	+	-	+
TMT label	126	127N	127C	128N	128C	129N	129C	130N	130C	131

TMT 10plex labeling set 2

**Table:** 

Temperature / °C	67.0	67.0	59.2	59.2	53.3	53.3	47.1	47.1	41.2	41.2
Compound treatment	-	+	-	+	-	+	-	+	-	+
TMT label	126	127N	127C	128N	128C	129N	129C	130N	130C	131

This approach is similar to the previously described RTSA approach [33], however, allowing for ten instead of nine temperature treatments by omitting common reference temperature samples in the two TMT sets and, instead, using a global melting curve fitting procedure described below.

#### LC–MS/MS analysis

In total, eight (high pH fractions per TMT set) x two (TMT sets per temperature; 37 °C and 50 °C) x three (replicates) = 48 TPP-CCR and eight (high pH fractions per TMT set) x two (TMT sets per replicate) times three (replicates) = 48 TPP-TR MS samples were analyzed using a Rapid Separation Liquid Chromatography System (Ultimate 3000, Thermo Fisher) and a nano-source ESI interface equipped Orbitrap Fusion Lumos Tribrid mass spectrometer (Thermo Fisher Scientific, Dreieich, Germany) operated in synchronous precursor selection (SPS) [47] mode as described [40].

#### MS data analysis, protein identification, and quantification

MS data was processed as described [40] using the MaxQuant software (Max Planck Institute for Biochemistry, Planegg, Germany) version 1.6.17.0 based on 75,777 Homo sapiens protein entries, downloaded from the UniProtKB on 27 January 2021, yielding protein quantifications by TMT reporter ions at the MS3 level for a total of 5992 and 4590 identified protein groups for TPP-CCR and TPP-TR, respectively (including potential contaminants, reverse hits and only by site identifications). In the following, for simplicity and readability, a MaxQuant "protein group" is referred to as "identified protein", "protein ID", or just "protein," and a representative protein for the protein group is selected.

#### Statistical analysis of melting curves

Statistical data analysis was performed using the R programming language (R version 4.1.2 (2021–11-01) on a x86_64-w64-mingw32/ × 64 (64-bit) platform). For TPP-CCR, data normalization, dose response curve fitting, and pseudo-R^2^ calculation was performed as described [40] with the different starting values for pEC50 (9.5, 8.2, 6.8, 5.5) and H (-3, -0.33, 0.33, 3) for the first series of fits.

The nls() or nlsLM() functions of the R packages stats or minpack.lm, respectively, were used for non-linear melting curve fitting as detailed below. The procedure consists of two main steps: 1) Preparation of TPP-TR data, 2) RTSA analysis.

##### Preparation of TPP-TR data by a three-step normalization procedure

**Normalization for each temperature** For each treatment temperature, the reporter ion intensities of the two (treatment and control) x three (replicates) = six samples were normalized such that when each sample was compared to a (selected) reference sample, the median of the logarithmized (log_2_) fold changes (*FC*s, i.e., the ratios) of all protein reporter ion intensities (median log_2_*FC*s) was zero. The sample to which the other samples had the highest number of positive median log_2_*FC*s was selected as the reference sample within each temperature sample set. The intensities of the other samples were adjusted by multiplying by the calculated constant $c=2^{\left(\text{median}\;\log_2FCs\right)}$. This initial normalization step accounts for pipetting errors and unnoticed differences in the number of cells used and makes the reasonable assumption that the vast majority of proteins are unaffected by treatment with the compound. Note that henceforth all protein reporter ion intensity ratios between the corresponding treatment and control samples (same temperature and replicate) remain unaffected, allowing for differential protein abundance analysis.

**Normalization to a global melting curve** For each of the ten temperatures, the mean of a set of 36 median log_2_*FC*s was determined by pairwise comparison of all six samples of the given temperature with all six samples of the lowest temperature (*T*_low_ = 36.5 °C), which served as a reference. These obtained intensity means for each of the ten temperatures, *I*(*T*), were plotted on the linear scale $\left(I\left(T\right)=2^{\text {mean}\left(\text {median}\;\log_2FCs\left(T\;\text {vs.}\;T_{\text {low}}\right)\right)}\right)$ against their corresponding temperatures, *T*, and a melting curve (Eq. 1) [40]) was fitted to the data points using the parameters *I*_min_ and *I*_max_ for the asymptotic intensity minimum and maximum (plateaus), respectively, as well as *T*_*m*_ and *s* as parameters for melting point and slope, respectively. Equation 1 was derived from Eq. 2 with *T*_*m*_ = *a*/*b* and *s* =  − *b*^2^/*a* and Eq. 2 originates from the original three parameter (*I*_max_ = 1, using normalized data) description [62] considering that four parameters (*I*_max_ as fitting parameter) have been shown to be more appropriate [48].

1 $$I(T) = I_{\text{min}} + (I_{\text{max}} - I_{\text{min}}) / \{1 +\text{exp}[(T_{m}/T - 1) s T_{m}]\}$$

2 $$I(T) = I_{\text{min}}+ (I_{\text{max}}- I_{\text{min}}) / \{1 +\mathrm{exp}[-(a/T - b)]\}$$

Each set of six samples per temperature point was then shifted in the direction of the global melting curve by multiplying it by a constant *c*(*T*), which was the ratio between the fitted value, *I*_fit_(*T*), and the value of the data point *I*(*T*) (*c*(*T*) = *I*_fit_(*T*)/*I*(*T*)) at that temperature. Analogous to the previous step (normalization of samples **within** each temperature treatment), the present step normalizes samples **between** temperature treatments to account for pipetting errors and differences in cell number. It is important to note that the present normalization step does not affect the ratios of the six samples **within** a temperature treatment (normalized in the previous step), because these six samples are all multiplied by the same constant. Also note that all single protein reporter ion intensity ratios within each TMT 10plex labeling set stay unaffected from here on, resulting in six pairs (three replicates of TMT 10plex labeling set 1 and three replicates of TMT 10plex labeling set 2, each comprising treatment and control) of melting curve data per protein.

**Normalization of protein-wise melting curves from each TMT 10plex labeling set** First, a rough leveling of the six pairs of melting curve data (six labeling sets) was performed for each protein individually by multiplying all protein intensities in a labeling set by the ratio between the maximum intensity of that protein across all sets and the maximum intensity of that protein in that set. Melting curves (Eq. 1) for each protein were then fitted to the combined data from all six labeling sets using four parameters (*I*_min_, *I*_max_, *T*_*m*_, and *s*) or, as failure alternative, three parameters (*I*_max_, *T*_*m*_, and *s*; *I*_min_ = 0). The fitted parameters (*I*_min_, *I*_max_, *T*_*m*_, and *s*) were retained as constants in the next fits to determine the scaling factors, *sf*_*i*_, for each of the six TMT-10plex labeling sets according to (Eq. 3), where *i* denotes labeling sets 1 through 6.

3 $$I_{i}(T) = sf_{i} \{ I_{\text{min}}+ (I_{\text{max}}- I_{\text{min}}) / \{1 +\mathrm{exp}[(T_{m}/T - 1) s T_{m}]\} \}$$

After applying the scaling to the six individual label sets per protein, the process of melting curve fitting over the combined labeling sets and scaling the individual labeling sets by scaling factors derived from the second fitting with only the scaling factor as a free parameter was repeated two more times to approach self-consistency. It is emphasized that the intensity ratios within the labeling sets were unaffected, so that the information about the protein intensity ratios between treatment and control (fold changes, *FC*s) was preserved.

##### RTSA analysis

For determining the effects of prodigiosin treatment on both protein thermal stability and protein abundance, the normalized reporter intensity data were used as input for the RTSA software (R package v1.0, [33]), which was run without another normalization of the data and was slightly modified to accept the present input data (no common reference data in the two present TMT labeling sets and 36.5 °C instead of 37 °C as lowest temperature).

### Immunoblotting

For SDS PAGE and western blotting, cells were harvested by scraping, pelletized at 150 rcf and 4 °C for 5 min, washed with DPBS and quickly frozen in liquid nitrogen. Cells were lysed in lysis buffer (20 mM Tris–HCl, 150 mM NaCl, 500 µM EDTA, 1% [v/v] Triton X-100, 1 mM Na_3_VO_4_, 10 mM NaF, 2.5 mM Na_4_P_2_O_7_, 1X protease inhibitor cocktail [Roche, Basel, Switzerland, #4693132001]) for 30 min on ice and the lysates were cleared by centrifugation at 18,000 rcf and 4 °C for 15 min. Protein concentration was determined by Bradford assay and sample buffer was added (62.5 mM Tris, 8.6% [v/v] glycerol, 2% [w/v] SDS, 33.3 µg/mL bromophenol blue, 1% [v/v] β-mercaptoethanol). Samples were heated at 95 °C for 5 min and then equal amounts of protein (25 µg) were subjected to SDS–polyacrylamide gels. For CETSA analysis of TPP-TR samples, the protein concentration was determined by Pierce 660 nm Protein Assay and the protein concentration of the 36.5 °C treated sample of each treatment and replicate was used for the other nine samples of the respective sample set to display temperature-dependent total protein aggregation and precipitation. 20 µg of protein were supplemented with sample buffer (62.5 mM Tris, 8.6% [v/v] glycerol, 2% [w/v] SDS, 33.3 µg/mL bromophenol blue, 1% [v/v] β-mercaptoethanol) and heated at 95 °C for 5 min before loading on SDS–polyacrylamide gels. After separation by SDS-PAGE, proteins were transferred to PVDF membranes (Merck, Darmstadt, Germany, #IPFL00010), blocked with 5% milk powder in TBST and analyzed using the indicated primary antibodies followed by appropriate IRDye 800- or IRDye 680-conjugated secondary antibodies (LI-COR Biosciences, Lincoln, NE, USA). Fluorescence signals were detected using an Odyssey Infrared Imaging system (LI-COR Biosciences, Lincoln, NE, USA) and signals were quantified with Image Studio (LI-COR Biosciences, Lincoln, NE, USA). For GRASP55 CETSA analysis, differential melting curve analysis was performed using the RTSA software as described for TPP-TR using normalized quantitative immunoblot data as input. Normalization was performed by dividing each of the two (treatment and control) x three (replicates) = six data sets (ten temperatures each) by the respective fitted *I*_max_ of three parameter (*I*_max_, *T*_*m*_, and *s*; *I*_min_ = 0) melting curve fits (Eq. 1).

### Generation of knock-out cell lines

GRASP55 KO cells were generated using the CRISPR/Cas9 system developed by the Zhang lab [59]. Double-stranded DNA oligos (5´-CACCGTCGCAAAGCGTCGAGATCCC-3´, 3´-AAACGGGATCTCGACGCTTTGCGAC-5´), encoding guide RNAs (gRNAs) against the target gene were cloned into the Bbsl restriction site of pSpCas9(BB)-2A-GFP (PX458) vector gifted from Feng Zhang (Addgene plasmid #48,138; http://n2t.net/addgene:48138; RRID:Addgene_48138). Cells were transfected with the resulting vector by electroporation using the Amaxa® Cell Line Nucleofector® Kit R (Lonza, Basel, Switzerland, #VCA-1001) according to the manufacturer´s instructions. Four days after transfection, individual clones were generated by cell sorting for GFP positive cells. GRASP55 knockout was validated by immunoblotting, immunofluorescence and DNA sequencing. For GRASP55 sequencing, genomic DNA was isolated using the GeneJET Genomic DNA Purification Kit (Thermo Fisher Scientific, Waltham, MA, USA, #K0721) according to the manufacturer´s instructions. Genomic loci were amplified by PCR using the following primers: 5′-GGGAACGCGTCTGCATAAATC-3′, 3′-TCCAGCCCGTCCTCCTACAG-5′. After poly(A) tailing using a TAQ DNA Polymerase (New England Biolabs, Ipswich, MA, USA, #M0267), PCR products were cloned into the pCR™ 2.1-TOPO™ TA-vector using a TOPO™ TA Cloning™ Kit (Thermo Fisher Scientific, Waltham, MA, USA, #450,641). The DNA sequence of 20 clones was determined by Sanger Sequencing using the M13for standard sequencing primer (5´-TGTAAAACGACGGCCAG-3´) and the sequencing results were aligned with NCBI Reference Sequences of GRASP55 (https://www.ncbi.nlm.nih.gov/nuccore/NM_015530.4).

### Secretome analysis

For the preparation of secretomes, 2*10^5^ HeLa wt or HeLa GRASP55 KO cells per well were seeded in a 6-well plate (*n* = 5 replicates for 4 different groups). On the next day, the cells were washed three times with DPBS and three times with serum-free culture medium and afterwards incubated in serum-free culture medium containing 0.1% DMSO or 100 nM prodigiosin for 24 h. After incubation, the medium was collected, centrifuged at 1,000 × *g* and 4 °C for 10 min and filtered through a 0.2 µm Acrodisc syringe filter (VWR, Radnor, PA, USA, #514–4131). After adding protease inhibitor cocktail (Roche, Basel, Switzerland, #5,892,791,001), the samples were snap frozen in liquid nitrogen and stored at -80 °C. Proteins were prepared for mass spectrometric analysis by a modified single-pot, solid-phase-enhance sample preparation (SP3) method. Here, 450 µl of conditioned medium was mixed with 50 µl of an 1 M aqueous 2-[4-(2-hydroxyethyl)piperazin-1-yl]ethanesulfonic acid solution, 250 µg of an 1:1 mixture of Sera-Mag SpeedBeads GE45152105050250 and GE65152105050250 (Merck, Darmstadt, Germany) and 1.25 ml acetonitrile and incubated for 10 min under constant shaking. Beads were washed with 70% ethanol and acetonitrile, proteins reduced with 10 mM dithiothreitol for 45 min at 56 °C and alkylated by adding 50 mM iodoacetamide. Subsequently, acetonitrile was added up to a concentration of 70% and after a 10 min incubation, beads were washed once with 70% ethanol, two times with 80% ethanol and finally with acetonitrile once. Proteins were digested with 0.05 µg trypsin in 50 mM triethylammonium bicarbonate in water overnight and for additional 4 h with newly added 0.05 µg trypsin. Tryptic peptides were collected, vacuum-dried and desalted using solid phase extraction (Oasis HLB µElution, Waters) using the manufacturers protocol. Finally, the sample was reconstituted in 0.1% trifluoroacetic acid and half of the sample analyzed by mass spectrometry.

First, peptides were separated over 1 h on C18 material using an Ultimate 3000 Rapid Separation Liquid Chromatography system (RSLC, Thermo Fisher Scientific, Waltham, MA, USA) essentially as described [57] and second injected into a Fusion Lumos mass spectrometer (Thermo Fisher Scientific, Waltham, MA, USA) via a nano-source electrospray interface. The mass spectrometer was operated in data-independent positive mode. First, a survey scan was recorded in profile mode (resolution 60,000, scan range 380–985 m/z, maximum injection time 100 ms, AGC target 400,000) followed by fragment spectra collected in the orbitrap analyser from mass windows of 10 Dalton size from a precursor range of 380–980 m/z (resolution 15,000, scan range 145–1450 m/z, maximum injection time 40 ms, AGC target 100,000, higher energy collisional dissociation, 30% collision energy).

Data analysis was carried out with DIA-NN version 1.8.1 [16] using standard parameters if not stated otherwise. A spectral library for the search was generated from protein sequences including potential contaminants (from MaxQuant 2.1.0.0, Max Planck Institute for Biochemistry, Planegg, Germany) and 81,837 homo sapiens entries form the UniProt KB proteome section (UP000005640, downloaded on 12^th^ January 2023). Beside carbamidomethylation at cysteines as fixed modification, N-terminal methionine excision as well as methionine oxidation were considered as variable modifications.

Only proteins identified with at least two different peptides and 4 valid intensity values (MaxLFQ) in at least one experimental group were considered for further analysis. Missing values of log_2_ transformed normalized intensities were filled in with values drawn from a down-shifted normal distribution (0.3 standard deviations width, 1.8 standard deviations down-shift) and differences of group means calculated for following pairs: wt prodigiosin treated – wt DMSO treated, KO prodigiosin treated – KO DMSO treated. Data was further annotated by ontology information provide by Perseus version 1.6.6.0 (Max Planck Institute for Biochemistry, Planegg, Germany) and OutCyte [83]. Annotation dependent significant abundance changes of protein groups were analysed using 1D and 2D annotation enrichment [11] and respective differences visualized with split-violin plots with OriginPro 2020b.

### Immunofluorescence

For immunofluorescence microscopy, HeLa cells were seeded on glass coverslips in 24-well plates. After treatment, cells were fixed in 4% paraformaldehyde for 15 min on ice, quenched with 50 mM NH_4_Cl for 15 min and permeabilized with 50 µg/mL digitonin (Sigma-Aldrich, St. Louis, MO, USA, #D141) for 5 min. Fixed samples were blocked with 3% BSA (Roth, Karlsruhe, Germany, #8076) for 30 min or overnight and incubated with primary antibodies diluted in 3% BSA for 2 h. Samples were then washed three times with DPBS, incubated with the appropriate secondary antibodies diluted in 3% BSA for 30 min and washed three times with DPBS. Afterwards, cells were embedded in ProLong Glass Antifade Mountant (Thermo Fisher Scientific, Waltham, MA, USA, #P36980) containing 1 µg/mL DAPI (Roth, Karlsruhe, Germany, #6335.1). Images were recorded with an Axio Observer 7 fluorescence microscope (Carl Zeiss Microscopy, Oberkochen, Germany) using a 40x/1,4 Oil DIC M27 Plan-Apochromat objective (Carl Zeiss Microscopy, Oberkochen, Germany) and an ApoTome 2 (Carl Zeiss Microscopy, Oberkochen, Germany).

### Statistical analysis

All IC_50_ values were calculated using GraphPad Prism 7.01. For transmission electron microscopy quantification, at least 50 Golgis per treatment and cell line were quantified after blinding and randomization. Results for cisternae number and length are shown in boxplot diagrams and P values were determined by ordinary one-way ANOVA with Dunnett´s post hoc test and are shown in the diagrams. For immunofluorescence analyses, dots, nuclei and co-localization were quantified and analyzed using ImageJ 1.53c. A dot to nuclei ratio was calculated for each image to determine the average number of dots per cell, and dots per cell and dot size were normalized by dividing through the mean dot number/size of the solvent control. All macros used for quantifications are provided in Supplementary Table S3. At least 15 representative images from three biological replicates per experiment were analyzed. For all immunofluorescence analyses, results are shown in boxplot diagrams and P values were determined by ordinary one-way ANOVA with Dunnett´s post hoc test and are shown in the diagrams.

### Supplementary Information

**Additional file 1:** **Figure S1.** Schematic representation of the thermal proteome profiling temperature range (TPP-TR) workflow. HeLa wt cells were treated with 100 nM prodigiosin or DMSO for 6 h. After the incubation, cells were harvested, washed and aliquots of the cell suspensions were exposed to short (3 min) treatments at different temperatures in the range between 36.5 °C and 67 °C. Cells were lysed and the non-denatured protein fraction was recovered after centrifugation. Quantitative protein analysis was performed by immunoblotting (CETSA) or MS (TPP). For MS, proteins underwent tryptic digest and the resulting peptides were labeled using TMT 10plex. The samples were combined such that prodigiosin treated and corresponding control samples belonging to the same temperature were analyzed within the same TMT set (similarly as described before for RTSA), allowing for studying not only thermal stability but also abundance effects upon prodigiosin treatment.

**Additional file 2:** **Figure S2.** STRING protein–protein association network analysis of the 93 significant prodigiosin-affected proteins given by the RTSA analysis (see figure 3A; significant proteins given by the RTSA software are colored in green or red for positive or negative RTSA distance score, respectively). Protein stabilization or destabilization (with p-value < 0.05 cutoff) is indicated by green or red squares, respectively, to the upper left of the circles representing the proteins. Likewise, an in- or decrease in abundance (with p-value < 0.05 and abs(mean log_2_ ratio 36.5 °C) > 0.1 cutoffs) is indicated by green or red squares to the lower left. Prominent clusters are outlined in blue (KEGG:hsa01212, fatty acid metabolism, destabilized proteins), purple (KEGG:hsa04142, lysosome, protein abundance decrease), and orange (KEGG:hsa04140, autophagy - animal). Proteins related to the Golgi apparatus (GO:0005794), Golgi membrane (GO:0000139), or Golgi-associated vesicles (GO:0005798) are labelled by “g”, “m”, or “v”, respectively.

**Additional file 3:** **Figure S3.** Secretome analysis upon prodigiosin treatment. HeLa wt and GRASP55 knockout cells were incubated for 24 h in serum free medium with and without 100 nM prodigiosin (*n*=5 per group). The conditioned medium was harvested and proteins analyzed by quantitative data-independent mass spectrometry. Differences of mean values of log_2_ normalized intensities between prodigiosin and DMSO treated samples were analyzed for distribution changes associated with protein categories including gene ontology cellular component (GOCC) and OutCyte using an 1D annotation enrichment analysis. OutCyte predicts signal peptides (SP, potential classical secretion pathway), transmembrane regions and leaderless secretion candidate proteins. Positive scores indicate a shift to higher abundances of proteins of a certain protein category, q-values represent for multiple comparisons corrected *p*-values (Benjamini- Hochberg method).

**Additional file 4. Supplementary Table S1**.

**Additional file 5.** **Supplementary Table S2**.

**Additional file 6. Supplementary Table S3.** (Macros used for quantifications).

**Additional file 7. **Original, uncropped immunoblots of Fig. 3E, 5A, and 7A.

## Abbreviations

B4GALT1: β-1,4-galactosyltransferase 1
BafA_1_: Bafilomycin A_1_
BFA: Brefeldin A
CETSA: Cellular thermal shift assay
EC_50_: Half maximal effective concentration
ECM: Extracellular matrix
ER: Endoplasmic reticulum
GRASP55: Golgi reassembly stacking protein of 55 kDa
GRASP65: Golgi reassembly stacking protein of 65 kDa
IC_50_: Half maximal inhibitory concentration
LAMP1: Lysosomal-associated membrane protein 1
LIR: LC3-interacting region
(MAP1)LC3: (Microtubule-associated proteins 1A/1B) light chain 3,
MS: Mass spectrometry
MTT: 3-(4,5-Dimethylthiazolyl-2)-2,5-diphenyl-2*H-*tetrazoliumbromid
pEC_50_: Negative decadic logarithm of the half maximal effective concentration
SP: Signal peptide
SQSTM1: Sequestosome 1
TEM: Transmission electron microscopy
TGN46: *trans*-Golgi network glycoprotein 46
*T*_*m*_: Melting temperature
TMT: Tandem mass tag
TPP-CCR: Thermal proteome profiling compound concentration range
TPP-TR: Thermal proteome profiling temperature range
V-ATPase: Vacuolar-type H^+^-ATPase
