# Supplementary figures and images for: The Golgi stacking protein GRASP55 is targeted by the natural compound prodigiosin
Source: Cell Commun Signal. 2023 Oct 5;21:275. doi: 10.1186/s12964-023-01275-1 (PMC10552397; doi:10.1186/s12964-023-01275-1)

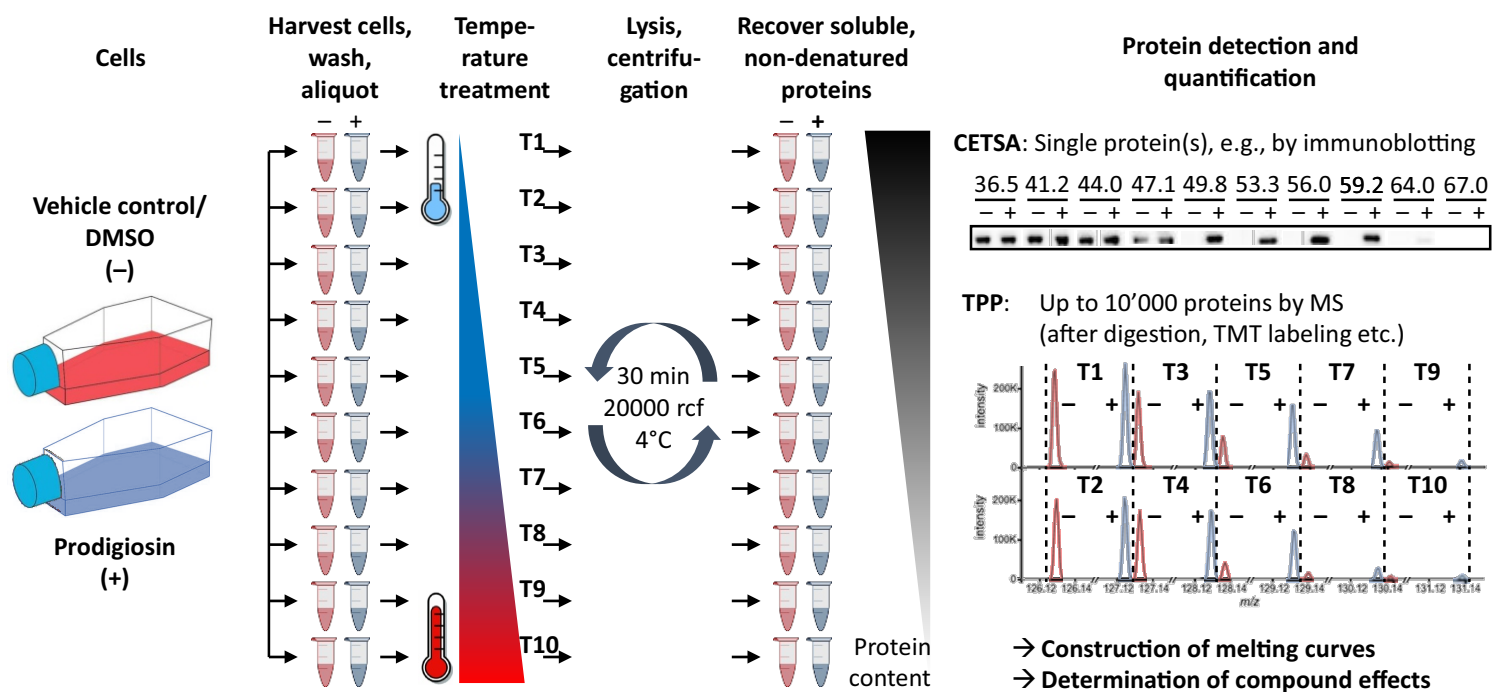

Figure S1

Supplement: Supplementary file 2 — Additional file 1: Figure S1. Schematic representation of the thermal proteome profiling temperature range (TPP-TR) workflow. HeLa wt cells were treated with 100 nM prodigiosin or DMSO for 6 h. After the incubation, cells were harvested, washed and aliquots of the cell suspensions were exposed to short (3 min) treatments at different temperatures in the range between 36.5 °C and 67 °C. Cells were lysed and the non-denatured protein fraction was recovered after centrifugation. Quantitative protein analysis was performed by immunoblotting (CETSA) or MS (TPP). For MS, proteins underwent tryptic digest and the resulting peptides were labeled using TMT 10plex. The samples were combined such that prodigiosin treated and corresponding control samples belonging to the same temperature were analyzed within the same TMT set (similarly as described before for RTSA), allowing for studying not only thermal stability but also abundance effects upon prodigiosin treatment. [file 12964_2023_1275_MOESM1_ESM.pdf]

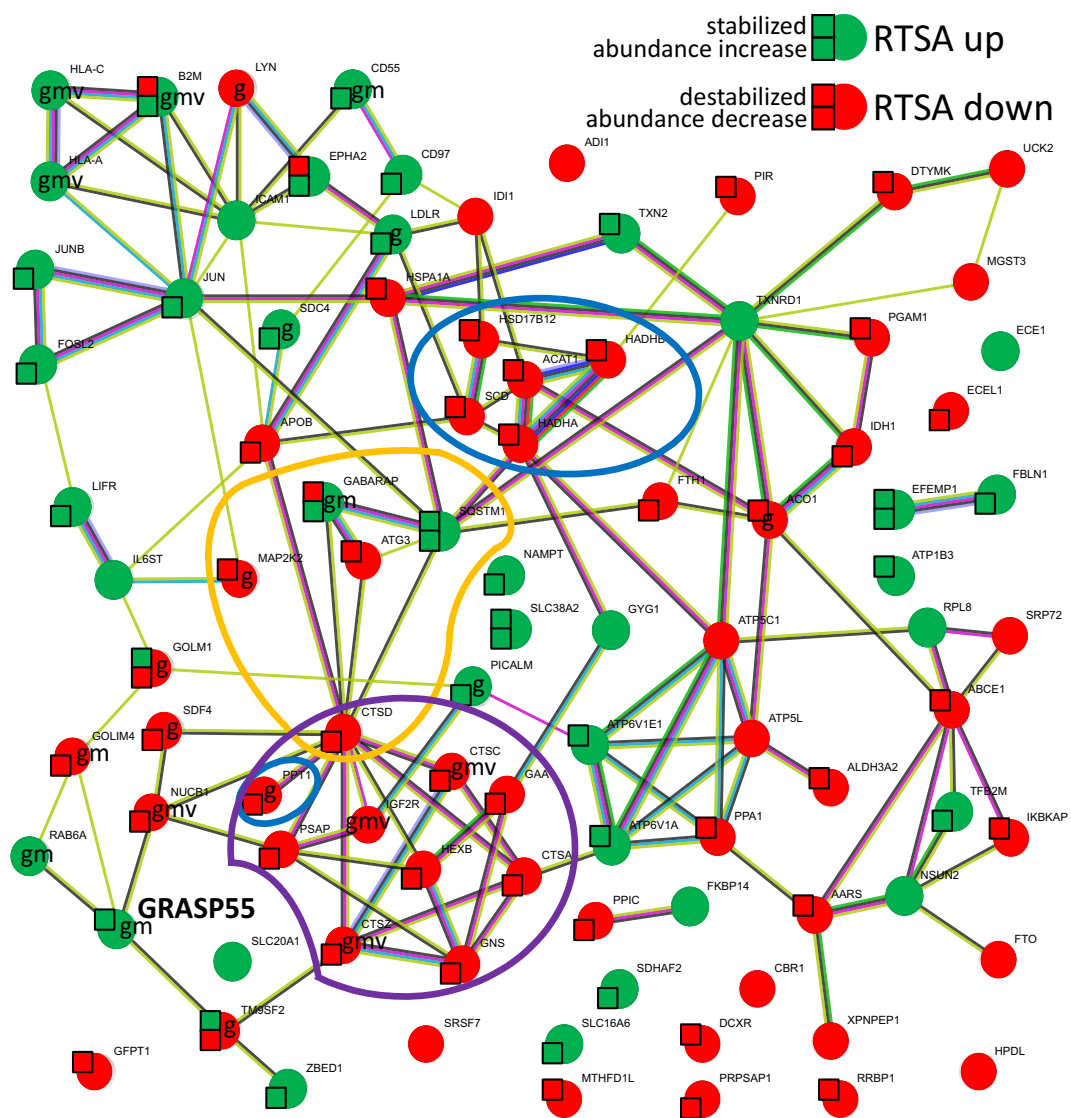

Figure S2

Supplement: Supplementary file 3 — Additional file 2: Figure S2. STRING protein–protein association network analysis of the 93 significant prodigiosin-affected proteins given by the RTSA analysis (see figure 3A; significant proteins given by the RTSA software are colored in green or red for positive or negative RTSA distance score, respectively). Protein stabilization or destabilization (with p-value < 0.05 cutoff) is indicated by green or red squares, respectively, to the upper left of the circles representing the proteins. Likewise, an in- or decrease in abundance (with p-value < 0.05 and abs(mean log2 ratio 36.5 °C) > 0.1 cutoffs) is indicated by green or red squares to the lower left. Prominent clusters are outlined in blue (KEGG:hsa01212, fatty acid metabolism, destabilized proteins), purple (KEGG:hsa04142, lysosome, protein abundance decrease), and orange (KEGG:hsa04140, autophagy - animal). Proteins related to the Golgi apparatus (GO:0005794), Golgi membrane (GO:0000139), or Golgi-associated vesicles (GO:0005798) are labelled by “g”, “m”, or “v”, respectively. [file 12964_2023_1275_MOESM2_ESM.pdf]

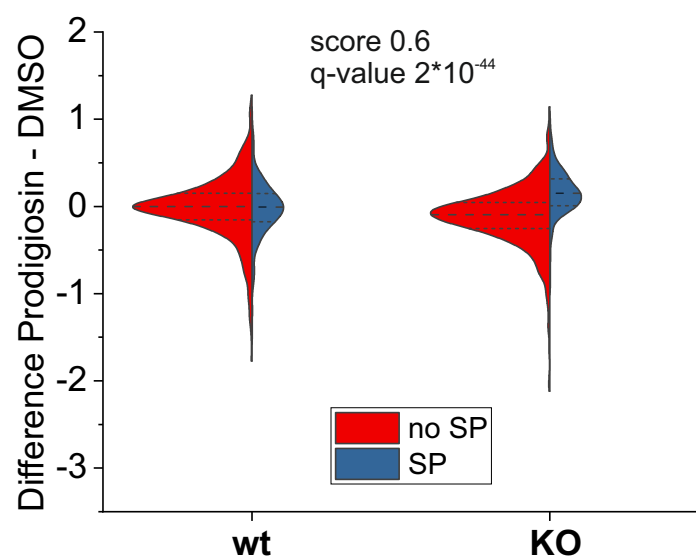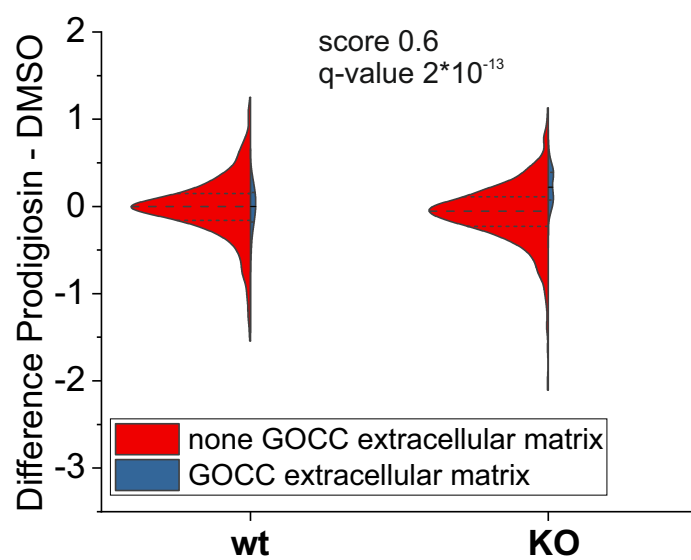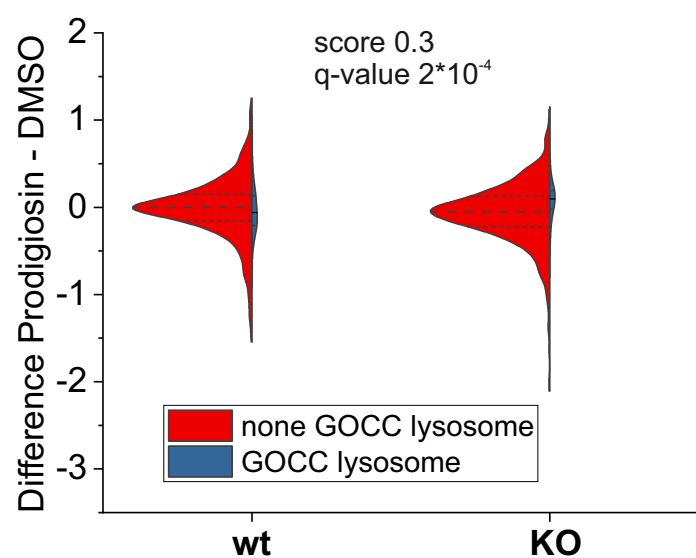

Figure S3

Supplement: Supplementary file 4 — Additional file 3: Figure S3. Secretome analysis upon prodigiosin treatment. HeLa wt and GRASP55 knockout cells were incubated for 24 h in serum free medium with and without 100 nM prodigiosin (n=5 per group). The conditioned medium was harvested and proteins analyzed by quantitative data-independent mass spectrometry. Differences of mean values of log2 normalized intensities between prodigiosin and DMSO treated samples were analyzed for distribution changes associated with protein categories including gene ontology cellular component (GOCC) and OutCyte using an 1D annotation enrichment analysis. OutCyte predicts signal peptides (SP, potential classical secretion pathway), transmembrane regions and leaderless secretion candidate proteins. Positive scores indicate a shift to higher abundances of proteins of a certain protein category, q-values represent for multiple comparisons corrected p-values (Benjamini- Hochberg method). [file 12964_2023_1275_MOESM3_ESM.pdf]

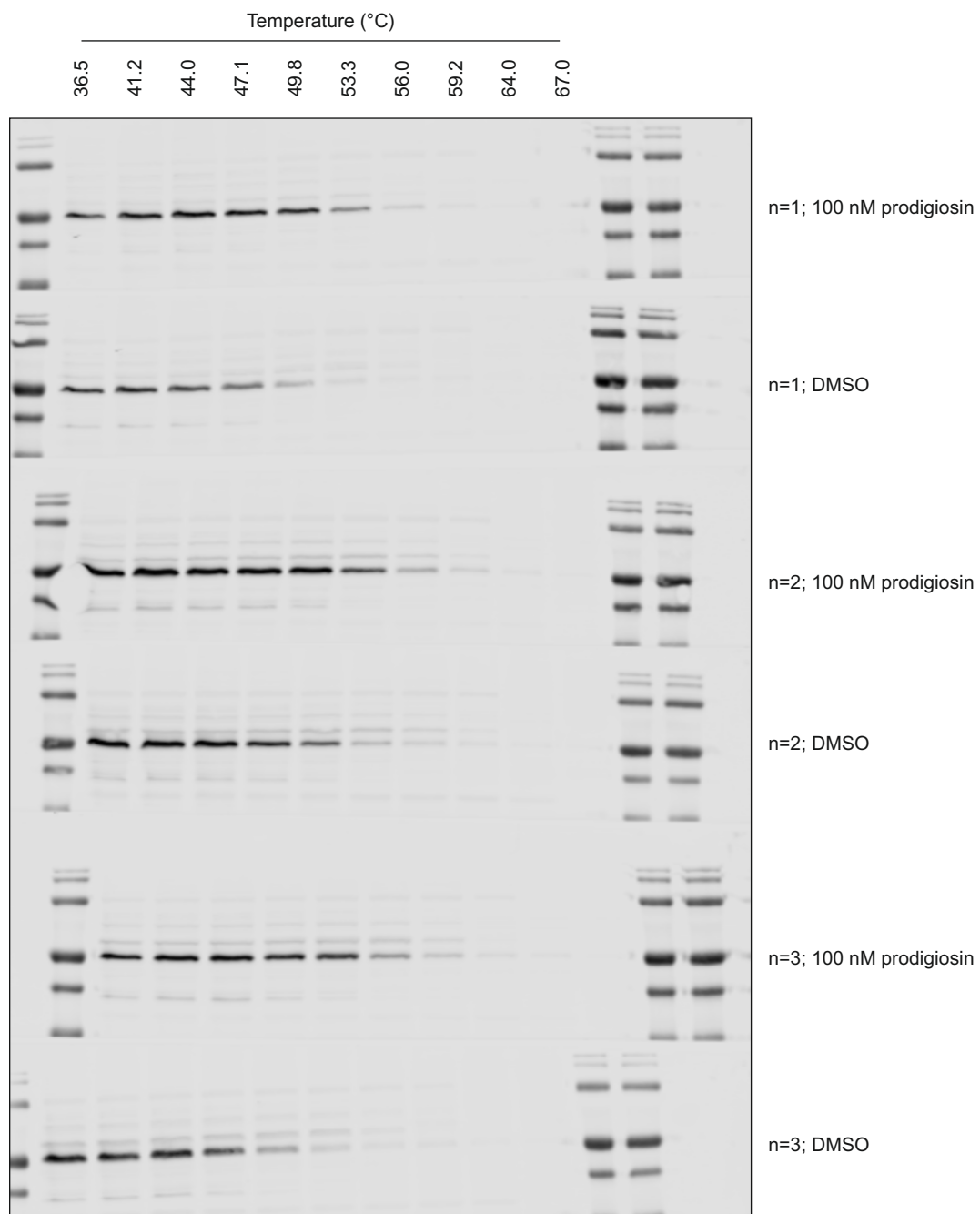

Figure 3

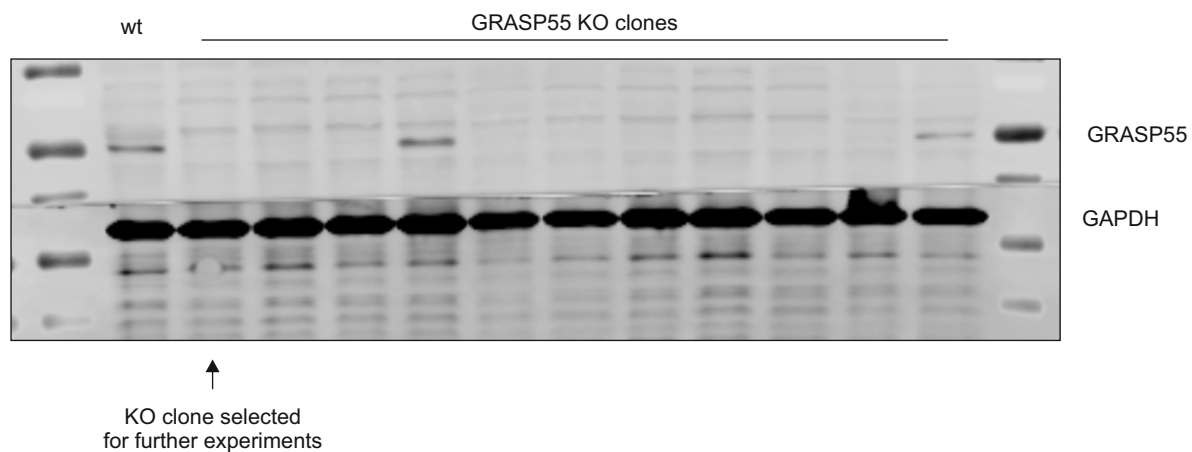

**Figure 5**

|   |   |   |   |   |   |                         |
|---|---|---|---|---|---|-------------------------|
| + | + | + | - | - | - | Full medium             |
| - | - | - | + | + | + | Starvation medium       |
| - | + | - | - | + | - | 100 nM Prodigiosin      |
| - | - | + | - | - | + | 10 nM BafA <sub>1</sub> |

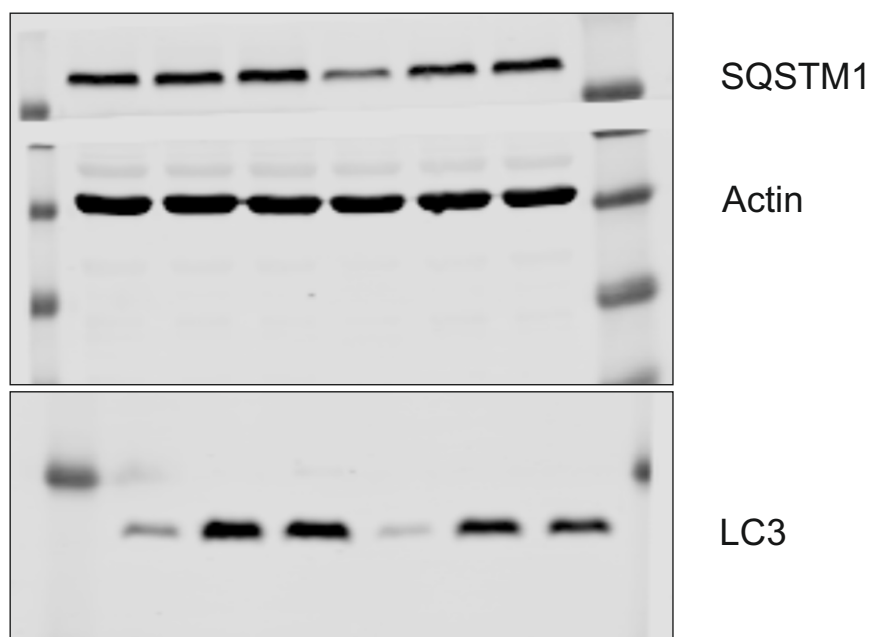

**Figure 7**

Supplement: Supplementary file 8 — Additional file 7. Original, uncropped immunoblots of Fig. 3E, 5A, and 7A. [file 12964_2023_1275_MOESM7_ESM.pdf]
