# Supplementary material for: The Golgi stacking protein GRASP55 is targeted by the natural compound prodigiosin
Source: Cell Commun Signal. 2023 Oct 5;21:275. doi: 10.1186/s12964-023-01275-1 (PMC10552397; doi:10.1186/s12964-023-01275-1)
Supplement: Supplementary file 7 — Additional file 6. Supplementary Table S3. (Macros used for quantifications). [file 12964_2023_1275_MOESM6_ESM.docx]

# Macros

## BFA washout assay:

### TGN46 quantification

dir1 = getDirectory("INPUT ");

dir2 = getDirectory("OUTPUT ");

setBatchMode(true);

list = getFileList(dir1);

for (i=0; i<list.length; i++)

{

open(dir1+list[i]);

title = File.nameWithoutExtension ;

//save channels

run("Z Project...", "projection=[Max Intensity]");

run("Split Channels");

close();

close();

run("Options...", "iterations=1 count=1 black do=Nothing");

run("Duplicate...", " ");

saveAs("Tiff", dir2+title+"_TGN46_roi.tif");

close();

run("8-bit");

saveAs("Tiff", dir2+title+"_TGN46.tif");

//process and count TGN46 puncta

run("Convoluted Background Subtraction", "convolution=Median radius=10");

run("Enhance Contrast...", "saturated=0.01 normalize");

run("Auto Threshold", "method=Moments white");

run("Adjustable Watershed", "tolerance=0.5");

saveAs("Tiff", dir2+title+"_TGN46_processed.tif");

run("Analyze Particles...", "size=0.01-Infinity show=Outlines exclude clear summarize add");

saveAs("Tiff", dir2+title+"_TGN46_drawing.tif");

close();

//select all ROIs in ROI manager

count=roiManager("count");

array=newArray(count);

for(f=0; f<count;f++)

array[f] = f;

roiManager("Select", array);

close();

//transfer ROIs to original image and measure intensities

open(dir2+title+"_TGN46_roi.tif");

roiManager("Show All");

roiManager("Measure");

run("8-bit");

saveAs("Tiff", dir2+title+"_TGN46_roi.tif");

//export to excel file on desktop

selectWindow("Results");

run("Read and Write Excel");

close("Results");

}

setBatchMode(false);

//save summary sheet

selectWindow("Summary");

saveAs("Text", dir2+"Summary.txt");

run("Close All")

showMessage("Mischief managed!");

exit();

### B4GALT1 quantification

dir1 = getDirectory("INPUT ");

dir2 = getDirectory("OUTPUT ");

setBatchMode(true);

list = getFileList(dir1);

for (i=0; i<list.length; i++)

{

open(dir1+list[i]);

title = File.nameWithoutExtension ;

//save channels

run("Z Project...", "projection=[Max Intensity]");

run ("Arrange Channels…", "new=213");

run("Split Channels");

close();

close();

run("Options...", "iterations=1 count=1 black do=Nothing");

run("Duplicate...", " ");

saveAs("Tiff", dir2+title+"_B4GALT1_roi.tif");

close();

run("8-bit");

saveAs("Tiff", dir2+title+"_B4GALT1.tif");

//process and count B4GALT1 puncta

run("Convoluted Background Subtraction", "convolution=Median radius=10");

run("Enhance Contrast...", "saturated=0.01 normalize");

run("Auto Threshold", "method=Moments white");

run("Adjustable Watershed", "tolerance=0.4");

saveAs("Tiff", dir2+title+"_B4GALT1_processed.tif");

run("Analyze Particles...", "size=0.01-Infinity show=Outlines exclude clear summarize add");

saveAs("Tiff", dir2+title+"_B4GALT1_drawing.tif");

close();

//select all ROIs in ROI manager

count=roiManager("count");

array=newArray(count);

for(f=0; f<count;f++)

array[f] = f;

roiManager("Select", array);

close();

//transfer ROIs to original image and measure intensities

open(dir2+title+"_B4GALT1_roi.tif");

roiManager("Show All");

roiManager("Measure");

run("8-bit");

saveAs("Tiff", dir2+title+"_B4GALT1_roi.tif");

//export to excel file on desktop

selectWindow("Results");

run("Read and Write Excel");

close("Results");

}

setBatchMode(false);

//save summary sheet

selectWindow("Summary");

saveAs("Text", dir2+"Summary.txt");

run("Close All")

showMessage("Mischief managed!");

exit();

### Nuclei quantification

dir1 = getDirectory("INPUT ");

dir2 = getDirectory("OUTPUT ");

setBatchMode(true);

list = getFileList(dir1);

for (i=0; i<list.length; i++)

{

open(dir1+list[i]);

title = File.nameWithoutExtension ;

run("Z Project...", "projection=[Max Intensity]");

run("Arrange Channels...", "new=312");

run("Split Channels");

saveAs("Tiff", dir2+title+"_DAPI.tif");

close();

close();

run("Unsharp Mask...", "radius=500 mask=0.90");

run("Median...", "radius=50");

run("Make Binary");

run("Fill Holes");

run("Adjustable Watershed", "tolerance=9");

run("Analyze Particles...", "size=6-Infinity summarize");

saveAs("Tiff", dir2+title+"_DAPI_counts.tif");

close();

}

setBatchMode(false);

//save summary sheet

selectWindow("Summary");

saveAs("Text", dir2+"Summary.txt");

run("Close All")

showMessage("Mischief managed!");

exit();

## GRASP55 and LC3 Co-Localization

### GRASP55 quantification

dir1 = getDirectory("INPUT ");

dir2 = getDirectory("OUTPUT ");

setBatchMode(true);

list = getFileList(dir1);

for (i=0; i<list.length; i++)

{

open(dir1+list[i]);

title = File.nameWithoutExtension ;

//save channels

run("Split Channels");

close();

close();

run("Options...", "iterations=1 count=1 black do=Nothing");

run("Duplicate...", " ");

saveAs("Tiff", dir2+title+"_GRASP55_roi.tif");

close();

run("8-bit");

saveAs("Tiff", dir2+title+"_GRASP55.tif");

//process and count GRASP55 puncta

run("Convoluted Background Subtraction", "convolution=Median radius=10");

run("Enhance Contrast...", "saturated=0.01 normalize");

run("Auto Threshold", "method=Moments white");

run("Adjustable Watershed", "tolerance=0.7");

saveAs("Tiff", dir2+title+"_GRASP55_processed.tif");

run("Analyze Particles...", "size=0.01-Infinity show=Outlines exclude clear summarize add");

saveAs("Tiff", dir2+title+"_GRASP55_drawing.tif");

close();

//select all ROIs in ROI manager

count=roiManager("count");

array=newArray(count);

for(f=0; f<count;f++)

array[f] = f;

roiManager("Select", array);

close();

//transfer ROIs to original image and measure intensities

open(dir2+title+"_GRASP55_roi.tif");

roiManager("Show All");

roiManager("Measure");

run("8-bit");

saveAs("Tiff", dir2+title+"_GRASP55_roi.tif");

//export to excel file on desktop

selectWindow("Results");

run("Read and Write Excel");

close("Results");

}

setBatchMode(false);

//save summary sheet

selectWindow("Summary");

saveAs("Text", dir2+"Summary.txt");

run("Close All")

showMessage("Mischief managed!");

exit();

### LC3 quantification

dir1 = getDirectory("INPUT ");

dir2 = getDirectory("OUTPUT ");

setBatchMode(true);

list = getFileList(dir1);

for (i=0; i<list.length; i++)

{

open(dir1+list[i]);

title = File.nameWithoutExtension ;

//save channels

run ("Arrange Channels…", "new=213");

run("Split Channels");

close();

close();

run("Options...", "iterations=1 count=1 black do=Nothing");

run("Duplicate...", " ");

saveAs("Tiff", dir2+title+"_LC3_roi.tif");

close();

run("8-bit");

saveAs("Tiff", dir2+title+"_LC3.tif");

//process and count LC3 puncta

run("Convoluted Background Subtraction", "convolution=Median radius=10");

run("Enhance Contrast...", "saturated=0.01 normalize");

run("Auto Threshold", "method=Moments white");

run("Adjustable Watershed", "tolerance=0.5");

saveAs("Tiff", dir2+title+"_LC3_processed.tif");

run("Analyze Particles...", "size=0.01-Infinity show=Outlines exclude clear summarize add");

saveAs("Tiff", dir2+title+"_LC3_drawing.tif");

close();

//select all ROIs in ROI manager

count=roiManager("count");

array=newArray(count);

for(f=0; f<count;f++)

array[f] = f;

roiManager("Select", array);

close();

//transfer ROIs to original image and measure intensities

open(dir2+title+"_LC3_roi.tif");

roiManager("Show All");

roiManager("Measure");

run("8-bit");

saveAs("Tiff", dir2+title+"_LC3_roi.tif");

//export to excel file on desktop

selectWindow("Results");

run("Read and Write Excel");

close("Results");

}

setBatchMode(false);

//save summary sheet

selectWindow("Summary");

saveAs("Text", dir2+"Summary.txt");

run("Close All")

showMessage("Mischief managed!");

exit();

### Nuclei quantification

dir1 = getDirectory("INPUT ");

dir2 = getDirectory("OUTPUT ");

setBatchMode(true);

list = getFileList(dir1);

for (i=0; i<list.length; i++)

{

open(dir1+list[i]);

title = File.nameWithoutExtension ;

run("Arrange Channels...", "new=312");

run("Split Channels");

saveAs("Tiff", dir2+title+"_DAPI.tif");

close();

close();

run("Unsharp Mask...", "radius=500 mask=0.90");

run("Median...", "radius=50");

run("Make Binary");

run("Fill Holes");

run("Adjustable Watershed", "tolerance=9");

run("Analyze Particles...", "size=6-Infinity summarize");

saveAs("Tiff", dir2+title+"_DAPI_counts.tif");

close();

}

setBatchMode(false);

//save summary sheet

selectWindow("Summary");

saveAs("Text", dir2+"Summary.txt");

run("Close All")

showMessage("Mischief managed!");

exit();

### Co-Localization

dir1 = getDirectory("INPUT ");

dir2 = getDirectory("OUTPUT ");

setBatchMode(true);

list = getFileList(dir1);

for (i=0; i<list.length; i++)

{

open(dir1+list[i]);

title = File.nameWithoutExtension ;

//split channels

getTitle();

run("Split Channels");

close();

selectWindow("C1-"+title+".czi - "+title+".czi #1");

rename("C1");

selectWindow("C2-"+title+".czi - "+title+".czi #1");

rename("C2");

run("JACoP ", "imga=[C1] imgb=[C2] pearson costesthr ica costesrand=10-1-1000-0.001-0-false-true-true");

close();

close();

close();

close();

close();

close();

close();

close();

}

setBatchMode(false);

selectWindow("Log");

saveAs("Text", dir2+"Summary.txt");

showMessage("Mischief managed!");

## LAMP1 and LC3 Co-Localization

### LAMP1 quantification

dir1 = getDirectory("INPUT ");

dir2 = getDirectory("OUTPUT ");

setBatchMode(true);

list = getFileList(dir1);

for (i=0; i<list.length; i++)

{

open(dir1+list[i]);

title = File.nameWithoutExtension ;

//save channels

run("Split Channels");

close();

close();

run("Options...", "iterations=1 count=1 black do=Nothing");

run("Duplicate...", " ");

saveAs("Tiff", dir2+title+"_LAMP1_roi.tif");

close();

run("8-bit");

saveAs("Tiff", dir2+title+"_LAMP1.tif");

//process and count LAMP1 puncta

run("Convoluted Background Subtraction", "convolution=Median radius=10");

run("Enhance Contrast...", "saturated=0.01 normalize");

run("Auto Threshold", "method=Moments white");

run("Adjustable Watershed", "tolerance=0.5");

saveAs("Tiff", dir2+title+"_LAMP1_processed.tif");

run("Analyze Particles...", "size=0.01-Infinity show=Outlines exclude clear summarize add");

saveAs("Tiff", dir2+title+"_LAMP1_drawing.tif");

close();

//select all ROIs in ROI manager

count=roiManager("count");

array=newArray(count);

for(f=0; f<count;f++)

array[f] = f;

roiManager("Select", array);

close();

//transfer ROIs to original image and measure intensities

open(dir2+title+"_LAMP1_roi.tif");

roiManager("Show All");

roiManager("Measure");

run("8-bit");

saveAs("Tiff", dir2+title+"_LAMP1_roi.tif");

//export to excel file on desktop

selectWindow("Results");

run("Read and Write Excel");

close("Results");

}

setBatchMode(false);

//save summary sheet

selectWindow("Summary");

saveAs("Text", dir2+"Summary.txt");

run("Close All")

showMessage("Mischief managed!");

exit();

### LC3 quantification

dir1 = getDirectory("INPUT ");

dir2 = getDirectory("OUTPUT ");

setBatchMode(true);

list = getFileList(dir1);

for (i=0; i<list.length; i++)

{

open(dir1+list[i]);

title = File.nameWithoutExtension ;

//save channels

run ("Arrange Channels…", "new=213");

run("Split Channels");

close();

close();

run("Options...", "iterations=1 count=1 black do=Nothing");

run("Duplicate...", " ");

saveAs("Tiff", dir2+title+"_LC3_roi.tif");

close();

run("8-bit");

saveAs("Tiff", dir2+title+"_LC3.tif");

//process and count LC3 puncta

run("Convoluted Background Subtraction", "convolution=Median radius=10");

run("Enhance Contrast...", "saturated=0.01 normalize");

run("Auto Threshold", "method=Moments white");

run("Adjustable Watershed", "tolerance=0.4");

saveAs("Tiff", dir2+title+"_LC3_processed.tif");

run("Analyze Particles...", "size=0.01-Infinity show=Outlines exclude clear summarize add");

saveAs("Tiff", dir2+title+"_LC3_drawing.tif");

close();

//select all ROIs in ROI manager

count=roiManager("count");

array=newArray(count);

for(f=0; f<count;f++)

array[f] = f;

roiManager("Select", array);

close();

//transfer ROIs to original image and measure intensities

open(dir2+title+"_LC3_roi.tif");

roiManager("Show All");

roiManager("Measure");

run("8-bit");

saveAs("Tiff", dir2+title+"_LC3_roi.tif");

//export to excel file on desktop

selectWindow("Results");

run("Read and Write Excel");

close("Results");

}

setBatchMode(false);

//save summary sheet

selectWindow("Summary");

saveAs("Text", dir2+"Summary.txt");

run("Close All")

showMessage("Mischief managed!");

exit();

### Nuclei quantification

dir1 = getDirectory("INPUT ");

dir2 = getDirectory("OUTPUT ");

setBatchMode(true);

list = getFileList(dir1);

for (i=0; i<list.length; i++)

{

open(dir1+list[i]);

title = File.nameWithoutExtension ;

run("Arrange Channels...", "new=312");

run("Split Channels");

saveAs("Tiff", dir2+title+"_DAPI.tif");

close();

close();

run("Unsharp Mask...", "radius=500 mask=0.90");

run("Median...", "radius=10");

run("Make Binary");

run("Fill Holes");

run("Adjustable Watershed", "tolerance=9");

run("Analyze Particles...", "size=6-Infinity summarize");

saveAs("Tiff", dir2+title+"_DAPI_counts.tif");

close();

}

setBatchMode(false);

//save summary sheet

selectWindow("Summary");

saveAs("Text", dir2+"Summary.txt");

run("Close All")

showMessage("Mischief managed!");

exit();

### Co-Localization

dir1 = getDirectory("INPUT ");

dir2 = getDirectory("OUTPUT ");

setBatchMode(true);

list = getFileList(dir1);

for (i=0; i<list.length; i++)

{

open(dir1+list[i]);

title = File.nameWithoutExtension ;

//split channels

getTitle();

run("Split Channels");

close();

selectWindow("C1-"+title+".czi - "+title+".czi #1");

rename("C1");

selectWindow("C2-"+title+".czi - "+title+".czi #1");

rename("C2");

run("JACoP ", "imga=[C1] imgb=[C2] pearson costesthr ica costesrand=10-1-1000-0.001-0-false-true-true");

close();

close();

close();

close();

close();

close();

close();

close();

}

setBatchMode(false);

selectWindow("Log");

saveAs("Text", dir2+"Summary.txt");

showMessage("Mischief managed!");
